# Supplementary material for: Pan-African genome demonstrates how population-specific genome graphs improve high-throughput sequencing data analysis
Source: Nat Commun. 2022 Aug 4;13:4384. doi: 10.1038/s41467-022-31724-3 (PMC9352875; doi:10.1038/s41467-022-31724-3)
Supplement: Supplementary file 1 — Supplementary Information [file 41467_2022_31724_MOESM1_ESM.pdf]

Supplementary Document: Pan-African genome demonstrates how population-specific genome graphs improve high-throughput sequencing data analysis

H. Serhat Tetikol[1,*,†], Deniz Turgut[1,†], Kubra Narci[1,†], Gungor Budak[1,†], Ozem Kalay[1], Elif Arslan[1], Sinem Demirkaya-Budak[1], Alexey Dolgoborodov[1], Duygu Kabakci-Zorlu[1], Vladimir Semenyuk[1], Amit Jain[1], and Brandi N. Davis-Dusenbery[1]

[1]Seven Bridges Genomics, Charlestown, MA, USA
[†]These authors contributed equally to this work.
[*]serhat.tetikol@sevenbridges.com (corresponding author)

Supplementary Section 1 Graph construction

Supplementary Section 1.1 Graph Building Pipeline

This pipeline takes a number of input population variants and GRCh38 raw reference and outputs the final linear reference and the graph for downstream tools. Workflow has four main steps (see Figure 1a):

- **ALT processing:** takes GRCh38 linear reference in combination with the ALT contig mappings and produces a variant file for the ALT variants as well as the initial linear reference
- **Prepare inputs:** takes a number of input variants (VCF files) and performs the necessary initial process (filter, split, normalize) for merging.
- **Merge variants:** Combines variants from multiple sources and produces the intermediate graph VCF with required annotations
- **Filter variants:** Performs two filtering steps (Structural Variation filter and Multimap filter) on the intermediate graph VCF and generates the final linear reference and the graph VCF

ALT processing

This step produces the intermediate linear reference as well as the variations from ALT contigs Intermediate linear reference is a combination of primary chromosomes, unplaced and unlocalized contigs and the additional decoys defined for the reference. ALT variations are derived from alignments of ALT contigs onto the primary sequences

Linear reference Linear reference is constructed using primary chromosomes, unplaced and unlocalized contigs from the assembly and decoys (HS38D1 and EBV) defined for the reference. ALT and NOVEL contigs are excluded since they will be represented as variations on the primary chromosomes and FIX contigs are ignored.

ALT variant generation ALT variant generation starts with the GFF files provided by GRC. These files describe alignment of ALT contig with respect to primary chromosome in CIGAR format. Preliminary variations are obtained by going through the CIGARs and extracting differences between sequences. In some cases, ALT mapping might contain inverted sequence patches (see Figure 2) and alternative alignments of the subsequence of an ALT are defined for these regions. In these cases, the variants from primary alignment are ignored in these regions and alternative mapping is used instead.

Variant splitting Alignments provided by GRC are less greedy and often combine nearby variants into large events even if there are large chunks of identical sequences in between. For example, consider the alignment for chr5_GL339449.2_alt: M29 I623 I2085 I1169 D623 D2085 D1169 M5399... . Consecutive insertion and deletion events can be combined and the alignment can be simplified into M29 I3196 D3196 M53399.... The event I3196 D3196 represents a sequence replacement of 3196 bases from the primary sequence by the ALT sequence. However, the majority of two sequences are identical and this replacement can be reduced into 7 single base changes (SNPs) instead.

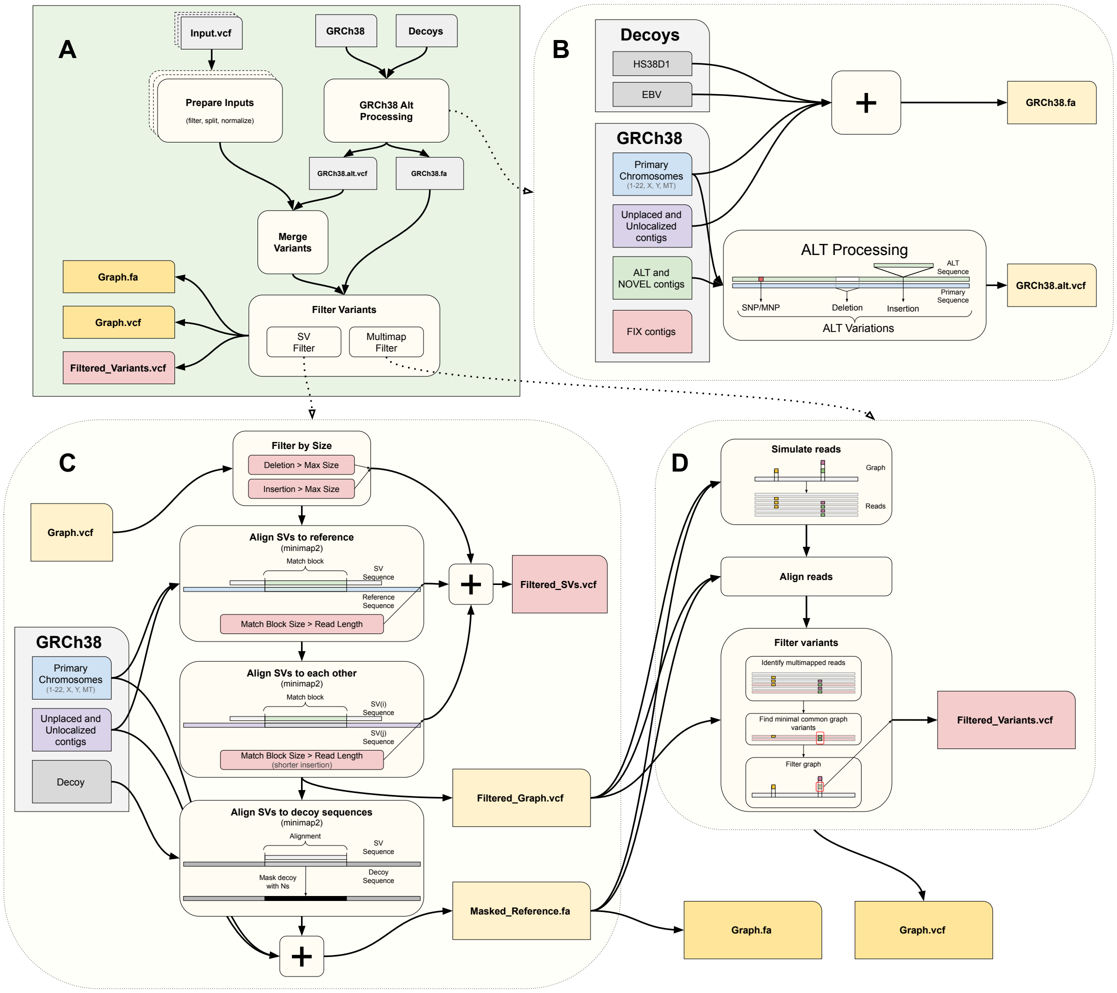

Supplementary Figure 1. Graph reference construction pipeline. The pipeline takes a set of VCF files and the linear reference (to be used as the backbone of the graph reference) as input, and outputs the constructed graph reference along with the modified linear reference and a list of variants that are excluded from the graph. (i) The input variants are processed to avoid any incompatibility with the graph representation and resolve potential issues in the VCF files. (ii) The alt-contigs in the linear assembly are added as edges into the graph reference and decomposed into smaller variants if necessary. (iii) All variants are merged and the allele frequency is re-calculated. (iv) Structural variants that are similar to the linear assembly or to each other are filtered and the decoy sequences are modified. (v) The reads are simulated from the constructed graph to detect and prune edges that cause multi-mapping in read alignment.

In order to obtain a more minimal representation of the variations, these kinds of replacement variations are aligned to each other using Needleman-Wunsch algorithm. If a long sequence of identical matching blocks are identified in the alignment then this replacement variation is split into smaller variations from the matching blocks.

Once all the variations for an ALT contig are obtained, they are left normalized with respect to the linear reference. Finally, variations from all the ALT contigs are merged and outputted as a VCF file.

Prepare inputs

Input preparation is a simple step to process VCF files from potentially different sources and unify the variant structure for merging. Steps involve:

- Split multiallelic variants
- Remove non-standard variant definitions and leaving only fully sequence resolved variants
- Apply any additional filtration steps (such as filter by allele frequency) to choose variants to be added

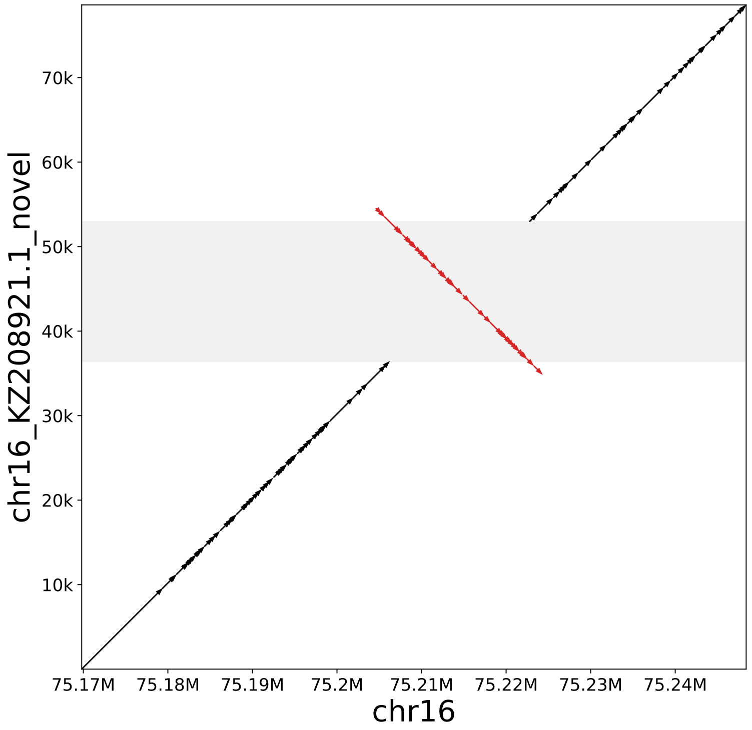

Supplementary Figure 2. Dot plot for an alt/novel contig Black arrows represent similarity in forward strand and red arrows represent similarity in reverse strand. This alt sequence contains an inverted subsequence (gray region) in the middle.

- Left normalize all variants to end up with consistent representation
- Clear unused annotations, ID and FILTER fields and sample information
- Annotate with necessary information to calculate effective allele frequency after merging
- Annotate variants to indicate the original source file using the ID assigned to the VCF file

Merge variants

Merge variants is a simple step that takes multiple prepared input VCF files and outputs a single biallelic graph candidate VCF file. It combines all variants into a single set while aggregating annotations if the same variant originates from multiple sourcesOnly additional processing is done to calculate the effective allele frequency if the same variation originates from multiple source files. Final allele frequency for a variation is the average of allele frequencies originating from all the source files weighted by the number of samples used for corresponding source file. Produced graph file is then pruned using the variant filtration step to obtain the final graph.

Variant filtration

Once the source variants are processed and merged, there needs to be some filtration applied in order to obtain a usable graph. Filtration process works in two steps. First, large events (structural variations) are handled in order to eliminate introducing sequences that would result in duplications. Second, remaining smaller variants are scanned for potential multimapping problems and problematic variants are pruned.

Structural variant filter Addition of large events (structural variations) may require special consideration for short read analysis. Especially, insertions longer than read size have to go through filtration in order to not make alignments ambiguous. Moreover, implementations of downstream tools can impose maximum size for these events. This filter aims to solve these problems and retain only useful structural variations in the graph. Flow of the process is shown in Figure 1c and details are as follows:

1. Events larger than defined maximum sizes are removed

2. Remaining insertions are aligned to the linear reference (using minimap2). If there is a subsequence (match block) in the insertion that is identical to the non-decoy sequences in the linear reference and is at least read length (default 150bp) size, adding that insertion would introduce ambiguity for some reads. That insertion is filtered.

3. Remaining insertions are aligned to each other (using minimap2). In similar fashion, if there is a common identical subsequence of at least read length, the smaller insertion is filtered.

4. Remaining insertions are added to the graph, but since decoys for a reference are obtained by common additional sequences that are not in the reference, it is possible that some of those sequences are already represented by the insertions and need to be taken out of the decoy. Therefore, remaining insertions are aligned to the decoy contigs and if there are alignments found, those regions on the decoy are masked with N bases.

Multimap filter As more edges are added to the graph, there is a risk of making some regions identical to other parts of the genome, therefore making alignment of reads ambiguous and uninformative. The goal of the multimap filter is to selectively remove graph edges such that the identity of regions is broken. Flow of the process is shown in Figure 1d and details are as follows:

1. Process starts with simulating short reads from all possible haplotypes on the graph. Genome is traversed at specified intervals for start positions and for a given start position, all possible paths of a specified length are generated as reads for that position. All the generated reads are collected as a FASTQ file.

2. Reads generated in the previous step are aligned using the graph

3. Resulting alignments are analyzed for potential bad edge or edge combinations
   (a) Reads at same starting position are grouped
   (b) Within the group there will be one linear reference only read and the rest will be reads following possible combinations of edges on the graph
   (c) Edges used for a non-reference reads is identified and that edge combination is classified as:
      - *BAD*: if read has a mapping quality of zero where reference read had mapping quality greater than zero
      - *GOOD*: if read has a mapping quality greater than the mapping quality of reference read or 20
      - ignored otherwise
   (d) If there are more than one classification of the same edge combination (i.e. different starting position), they are aggregated.
   (e) Edge combination is flagged for filtration if it has only *BAD* reads
   (f) A minimal subset of edges are identified from the flagged edge combinations, such that each flagged combination will have at least one common edge with the subset
   (g) Identified edges within the subset are removed from graph and final graph is outputted

Supplementary Section 1.2 Allele frequency cutoff experiments

To be able to obtain a more relevant and representative population-specific graph reference, we experimented with different allele frequency (AF) cutoffs to select the common variants to that population. We used Genome-in-a-Bottle (GIAB) Ashkenazim samples HG002, HG003 and HG004 and East Asian samples HG005, HG006 and HG007[1]. We then used gnomAD v3 variants to construct Ashkenazim (N=1,662) and East Asian (N=1,567) graph references at different AF cutoffs (0.5%, 1%, 5%, 10% and 20%)[2]. We ran the Seven Bridges GRAF Workflow for every sample and graph reference constructed to obtain alignment and variant calling results. We also included previous alignment and variant calling results for BWA+GATK and Seven Bridges GRAF using the public Seven Bridges Pan-Genome Graph Reference v1 to see the improvement in using population-specific graph reference compared to linear approach and global graph reference. We calculated comprehensive alignment quality control metrics including graph-specific metrics. We also used SB VCF Benchmark app to benchmark variant calling results against the sample truth sets and Mendelian Violation Detector app to determine the trio concordance among variant calling results.

The alignment QC results show that using graph references increases informative alignment (mapping quality or MAPQ ≥ 20), reduces multi-mapped (MAPQ$=0$) and uninformative (MAPQ<20) alignments, reduces improperly paired reads and error rate. The use of population-specific graph reference further improves alignment QC. There is an increase in the unmapped reads when we compare BWA+GATK and Pan-Genome; however, some of the increase can be avoided using population-specific graph references as the results show (Figure 3).

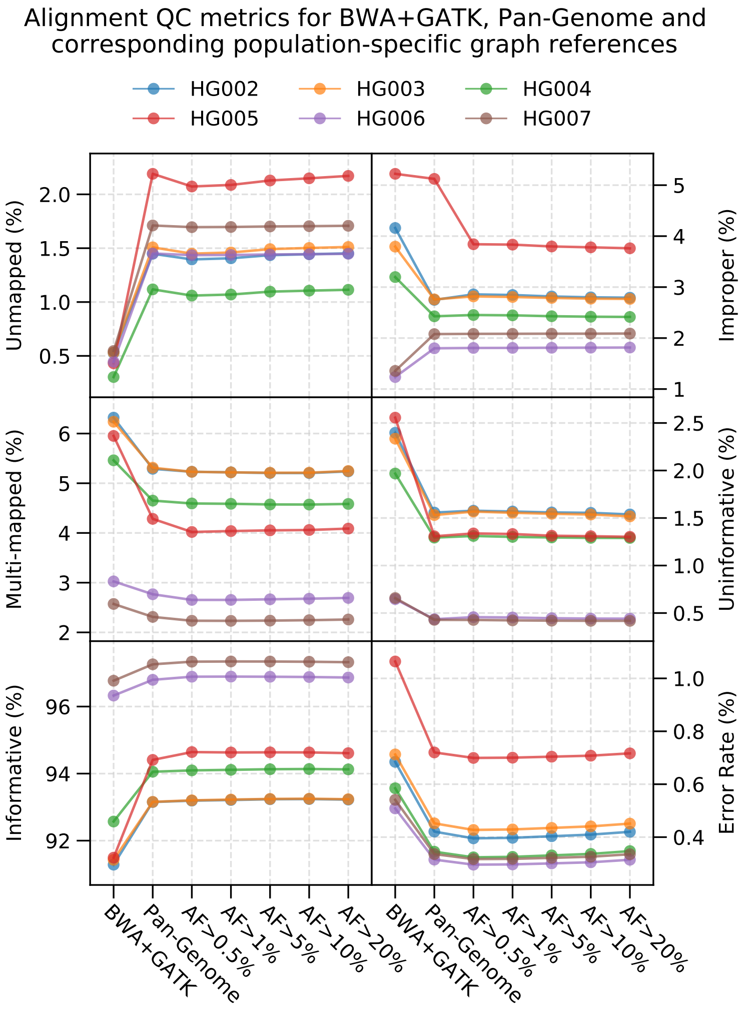

Supplementary Figure 3. **Alignment metrics for GIAB samples.**

The benchmark results show that with the graph references, there is a significant improvement in F-measure, reduction in the total error (the sum of false positives and false negatives) and the variants are more concordant within trios. The use of population-specific graph references further improves these metrics especially in SNP F-measure, SNP total error and INDEL discordance (Figure 4).

The overall results suggest that the use of population-specific graph references is better and the 5% allele frequency cutoff seems working the best for the majority of the metrics and it can be selected as a candidate for the future AF cutoff during the population-specific graph construction.

Supplementary Section 1.3 Content of the graph references used or constructed

We used the public Seven Bridges Pan-Genome Graph Reference v1 as the global graph reference and we constructed several population-specific graph references in this study[3]. The first population-specific graph was constructed by using only the public resources and the subsequent population-specific graph references were iteratively augmented with the common cohort variants to this graph with the public resources.

The public Seven Bridges Pan-Genome Graph Reference v1 (Pan-Genome) has been constructed using the GRCh38 human reference main contigs as the backbone and GRCh38 human reference alternative contigs, 1000 Genome Project common variants (N=2,504, AF> 1%), Simons Genome Diversity Project common variants (N=278, AC> 10) and the indels discovered by Mills et al (N=36, entire set) and 1000 Genomes consensus indels (N=2, entire set) were augmented to the main contigs in the graph[4–6]. The number of edges each source provides to the Pan-Genome graph is given in Supplementary Table 3.

The population-specific graph with the public resources (Pan-African-0) was constructed using the gnomAD v3 variants that are common in African population (N=21,042, AF_afr≥ 5%). These common African gnomAD v3 variants were augmented

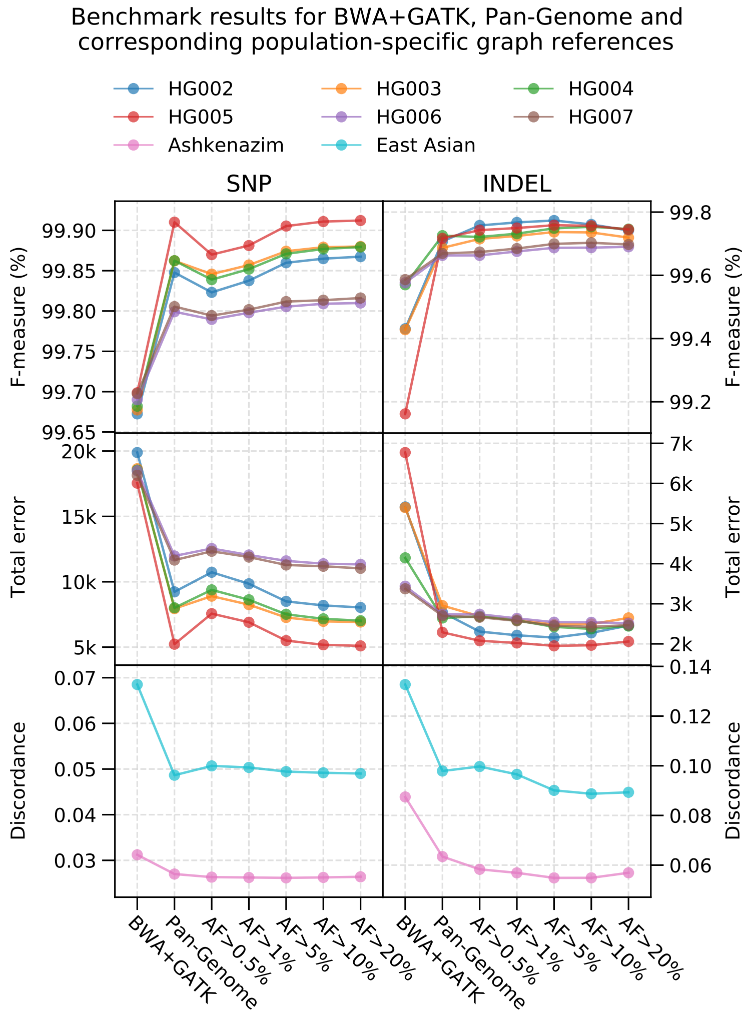

Supplementary Figure 4. Benchmark results for GIAB samples.

with GRCh38 human reference alternative contigs to the GRCh38 human reference main contigs as the backbone[2]. The iterative population-specific graph references (Pan-African-i where i={1, 2, 3, 4, 5}) were constructed using the resulting variant set at each previous group analysis (N=104, AF$\geq$ 5%) in addition to the sources used in the Pan-African-0 graph. List of samples in each group and the benchmarking set is provided in Supplementary Table 14. We also augmented the structural variants (N=10, entire set) discovered by the Human Genome Structural Variation Consortium (HGSVC) in the iterative population-specific graph references[7]. Moreover, we generated a non-iterative Pan-African graph using combined construction variant sets that are subsetted from the variant sets produced by NYGC using the 1000 Genomes high coverage datasets[8]. The number of edges each source provides to the population-specific graph references is given in Supplementary Table 3.

Contents of graph references are shown in Supplementary Table 2. Pan-African graphs include less SNPs than Pan-Genome graph but they contain more insertion and deletions, and difference further increases with addition of African SVs in Pan-African-1 stage. As new groups are included in the graph each type of variant slowly increased. While the Pan-Genome graph includes the more edges than some of the population-specific graphs, the average per sample use rate is lower. Out of 12.5 million SNPs only 35% of them are used in the benchmark set for Pan-Genome, but 43% of 12 million SNP edges are used in Pan-African 5. Used deletion edge rate is also 35% for Pan-Genome graph while it is 46% for Pan-African. The most used edge type is insertion, with 77% of Pan-Genome and 80% of Pan-African graph insertions are used in the benchmark set. A non-iterative graph constructed using BWA+GATK results is also compared to the other graphs. It includes the least amount of SNP edges and on average the use edge rate of SNPs is as high as population-specific graphs. However, since BWA+GATK graph contains cohort specific INDELs as well as the additional SVs, INDEL use edge rate is high similar to other population-specific graphs.

We look at the variant composition of the construction sets that are used to construct the graphs *Pan-African 1-5*. The comparison of variants found in each construction set is shown in Figure 5. It is seen that most of the common variants (around 94%) with $AF \geq 5\%$ are shared in all sets as expected, since all sets contain the same number of samples from each African population and the same female/male ratio. This implies that the processing order of these sets in the project workflow is unimportant. We also observe a significant reduction in the number of variants shared by subsequent groups and the mean AF of the variants added. This shows that most of the more common variations are captured in earlier iterations, and subsequent groups contribute mostly infrequent variants around AF cut-off. The total number of variants for each set is shown in the horizontal bars. A consistent increase is observed with each iteration. This indicates that the variant calling sensitivity increases with each graph augmentation and there is merit in augmenting graphs even during graph constructions.

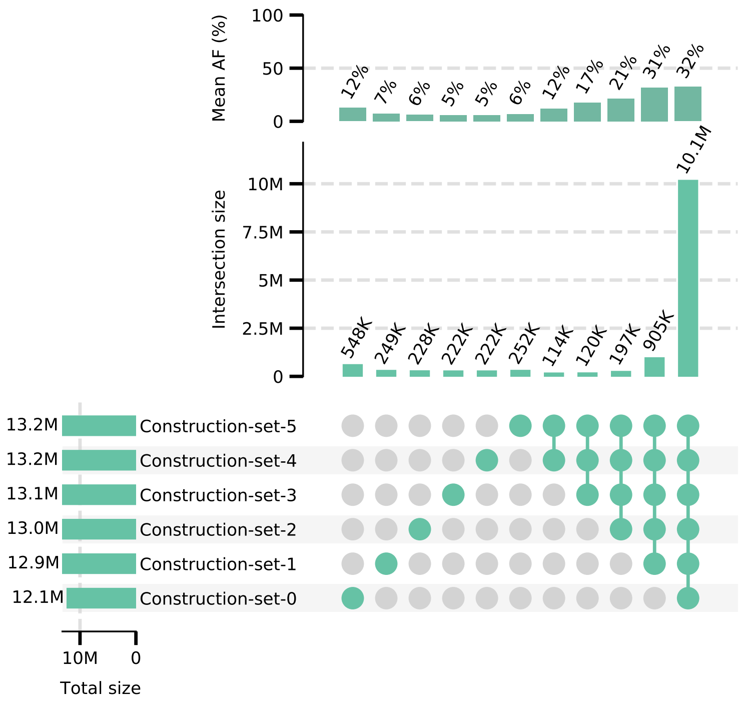

Supplementary Figure 5. Comparison of construction set variants.

In order to test the generalizability and the effectiveness of the Pan-African graph reference constructed in this study, we measured its performance on a set of independent African samples from the Human Genome Diversity Project (HGDP)[9]. This dataset contains samples from 7 African population groups (Mandenka, Yoruba, Biaka, Mbuti, San, Bantu/Kenya and Bantu/South Africa; full sample list is provided in Supplementary Table 15), only one of which is captured in the 1000 Genomes dataset. The results show that the Pan-African graph constitutes a more accurate representation of the African populations in the HGDP dataset compared to the Pan-Genome graph or the linear reference GRCh38, and therefore leads to more accurate alignment and more sensitive variant calling as shown in Figures 6 and 7. These results support the hypothesis that, even though 6 out of 7 African populations were not explicitly used to construct the Pan-African graph, we were able to capture the shared genetic background through other samples of African ancestry and public databases.

Finally, we constructed a Pan-Genome graph using gnomAD v3 variants based on a global allele frequency cutoff ($AF \geq 5\%$). The alignment and variant calling statistics are shown in figures 8 and 9. The sample composition of gnomAD database is significantly non-uniform. Almost half of the samples in the database are of European ancestry and nearly a quarter of them are African ancestry with the rest of the populations occupying remaining 25% of the samples. This skewed population distribution results in European and African ancestry populations being over-represented, while the other populations being under-represented. This can also be seen from the graph utilization metrics in 8G and H. gnomAD Pan-Genome is closer to African population graphs than a completely population agnostic graph would be, e.g. the original Pan-Genome graph we constructed. Eliminating this population bias in the gnomAD database would require a non-trivial normalization across allele frequencies of all populations. For this reason, we decided to not use gnomAD as a variant source for the Pan-Genome graph but only as a population-specific resource for the construction of Pan-African graphs.

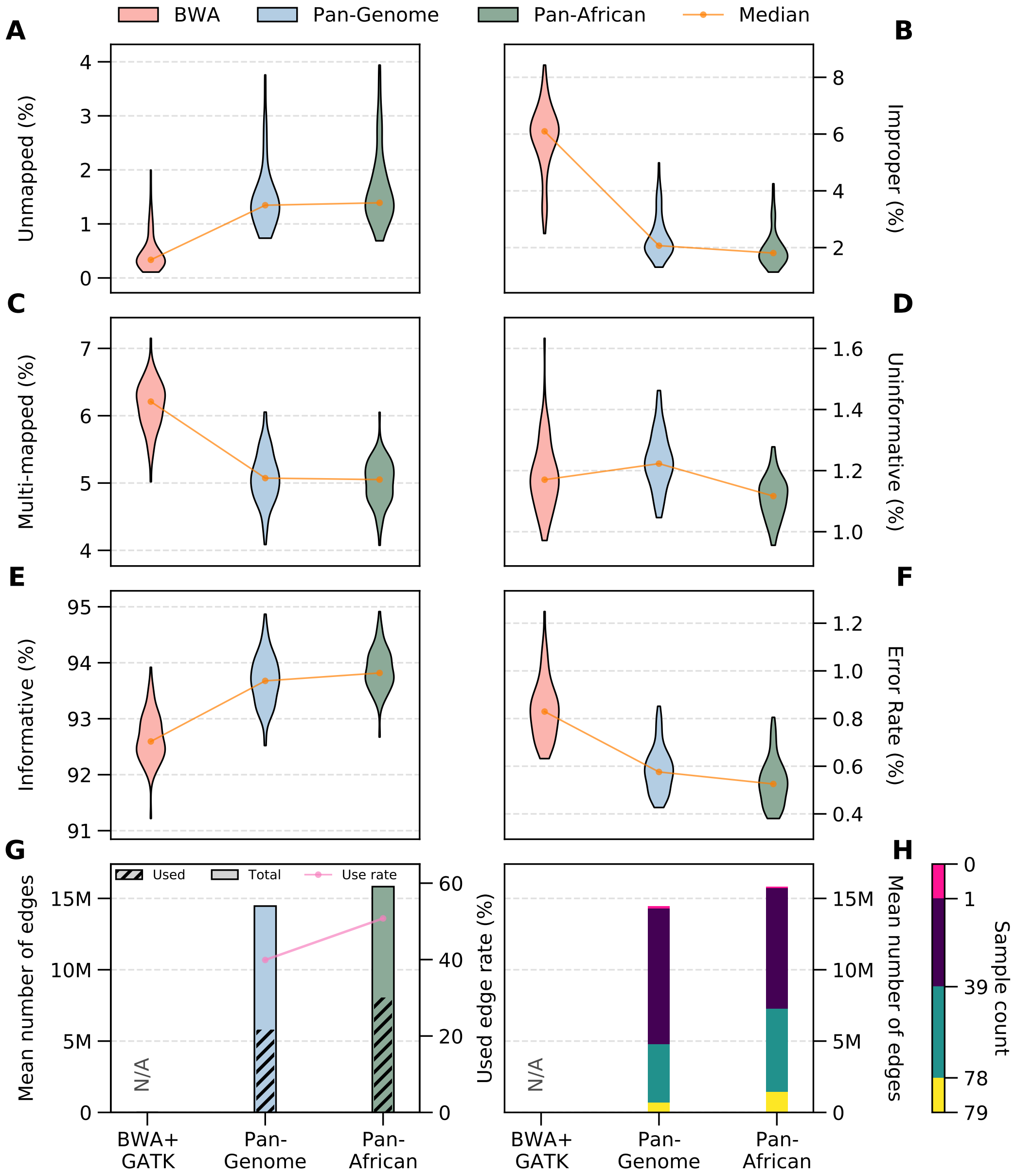

Supplementary Figure 6. Alignment metrics for BWA+GATK, Pan-Genome and Pan-African 5 graph pipelines measured on the African populations from the HGDP dataset. Two-sided Wilcoxon tests between consecutive distributions are performed. In all cases except for two (unmapped and multi-mapped between Pan-Genome and Pan-African) the difference is significant ($p < 10^{-7}$).

Supplementary Section 2 Nucleotide diversity and absolute divergence

We calculated the nucleotide diversity for all major super-populations and corresponding populations from the 1000 Genomes dataset. Results are shown in Figure 10. With the exception of American super-population, super-populations generally have similar diversity as their populations. America displays a varying diversity among the populations.

The TPR and FPR are also calculated theoretically, assuming an underlying AF distribution obtained empirically for each super-population, as shown in the inset (see Methods for details). The results are shown in Figure 11a. It is seen that the TPR and FPR improve with increasing number of samples albeit with diminishing returns for larger sample sizes. For instance, by constructing a graph using approximately 700 samples of African ancestry, one can expect to capture 98% of the variation information with a false positive rate slightly above 2%. The experiment and theory agree well, with some deviation for large number of samples due to the limited number of samples available in the experiment. It is important to note that the rate of

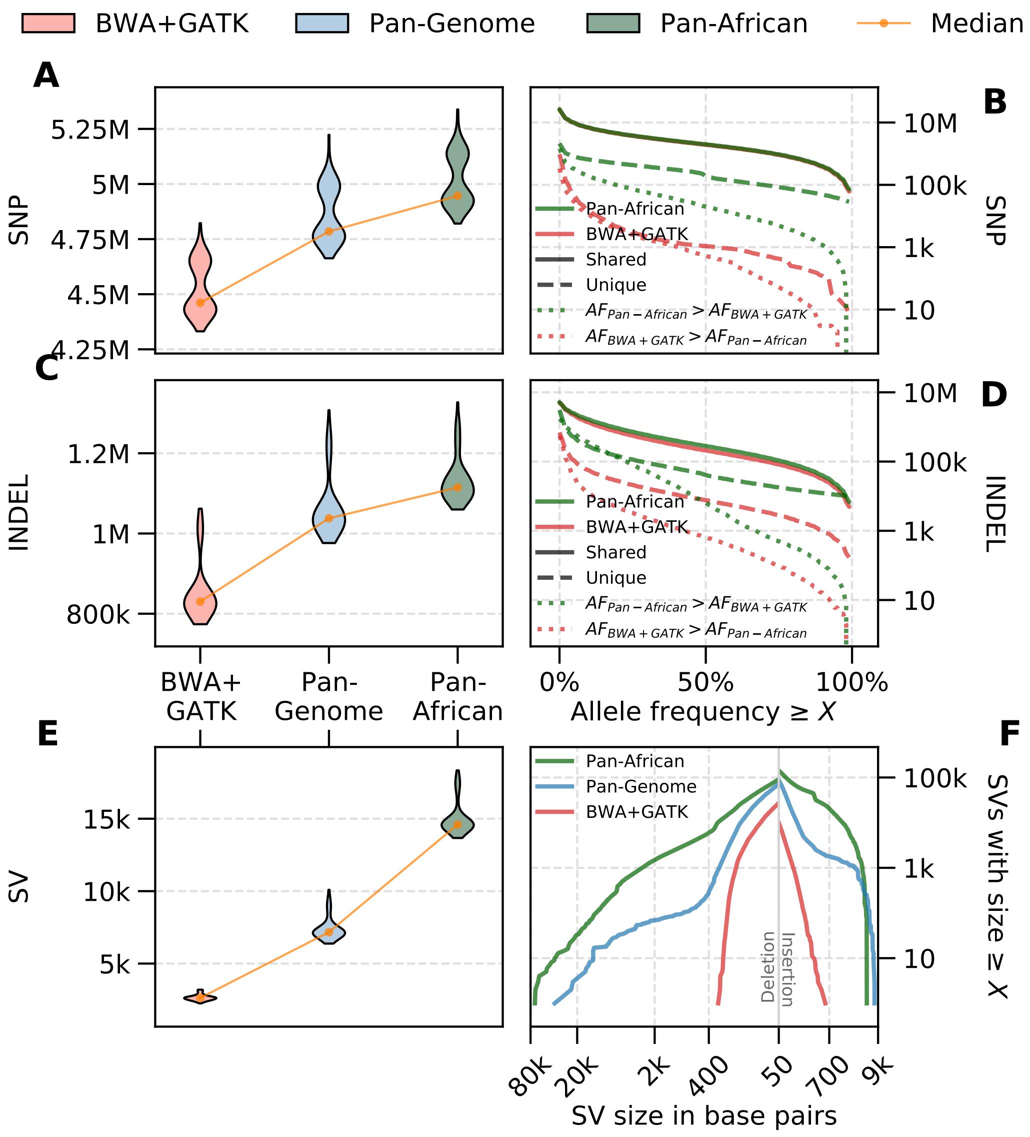

Supplementary Figure 7. Variant calling metrics for BWA+GATK, Pan-Genome and Pan-African 5 graph pipelines measured on the African populations from the HGDP dataset. Two-sided Wilcoxon tests between consecutive distributions are performed. In all cases the difference is significant ($p < 10^{-13}$).

improvement with increasing number of samples for each super-population is inversely correlated with the nucleotide diversity of the super-population (Figure 10). The descending order of nucleotide diversity is $AFR > AMR > SAS > EUR > EAS$, which is exactly the same order of improvement rates, i.e. convergence rates, from low to high. For instance, we observe a slower convergence for the African super-population as opposed to the European super-population because the former is much more diverse. The East Asian super-population graph, on the other hand, improves the fastest since it has the lowest nucleotide diversity. It is also noteworthy that the convergence rate does not depend on the absolute divergence from GRCh38, as mentioned earlier. This is illustrated by the East Asian TPR and FPR curves, which have the highest convergence rates even though the divergence of the East Asian super-population is higher than all super-populations except the African. A detailed table for different values of AF cutoff and TPR is provided for all super-populations along with the corresponding FPR in Supplementary Table 1. This table can be used as a guideline for other super-populations via a comparison of their nucleotide diversity.

In order to measure the effect of sampling on the graph convergence rates, we compare a *homogeneous* sampling approach to a *clustered* sampling approach. Homogeneous sampling ensures that samples for graph construction are picked from all

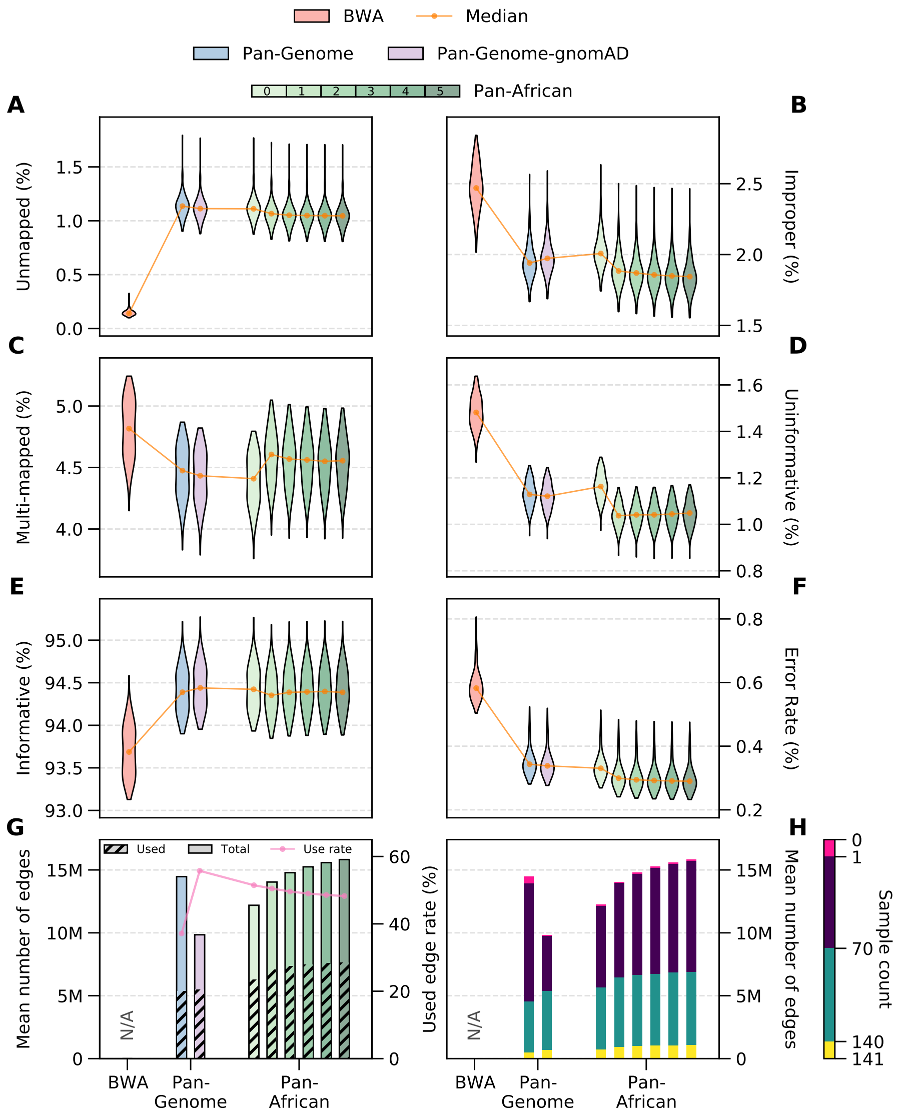

Supplementary Figure 8. Alignment metrics for graphs including gnomAD Pan-Genome graph. Two-sided Wilcoxon tests between gnomAD Pan-Genome and other distributions are performed. In all cases the difference is significant ($p < 10^{-19}$).

populations uniformly so that there are no abrupt changes in the genetic architecture of the graph reference as more samples are added. Although this approach is expected to more quickly capture the super-populations genetic information, it is not always possible due to missing detailed ancestry information or different populations being sequenced in different phases of the project. To compare homogeneous sampling to the worst case scenario, clustered approach assumes that the samples are first picked exclusively from a specific population until there are no samples left belonging to that population. Then, the procedure is applied to another population until all populations in the dataset are exhausted. The results are shown in Figure 11b. It is clear that clustered sampling results in a slower convergence rate; therefore, graph references are significantly less representative of the super-population compared to homogeneous sampling. Abrupt jumps are also observed in TPR and FPR curves at points where a new population is introduced to the graph reference.

We also calculated TPR and FPR rates for different cutoffs (AF$\geq$ 0.01, AF$\geq$ 0.10 and AF$\geq$ 0.20) in addition to the AF$\geq$ 0.05 shown in the main text. Results are shown in Figure 12. Expected number of samples and corresponding FPR rates are also calculated for various TPR rates and listed in Supplementary Table 1. Number of samples required to reach a target TPR follows the diversity for corresponding super-populations. The more diverse the population is, the more samples are required to get a representative graph.

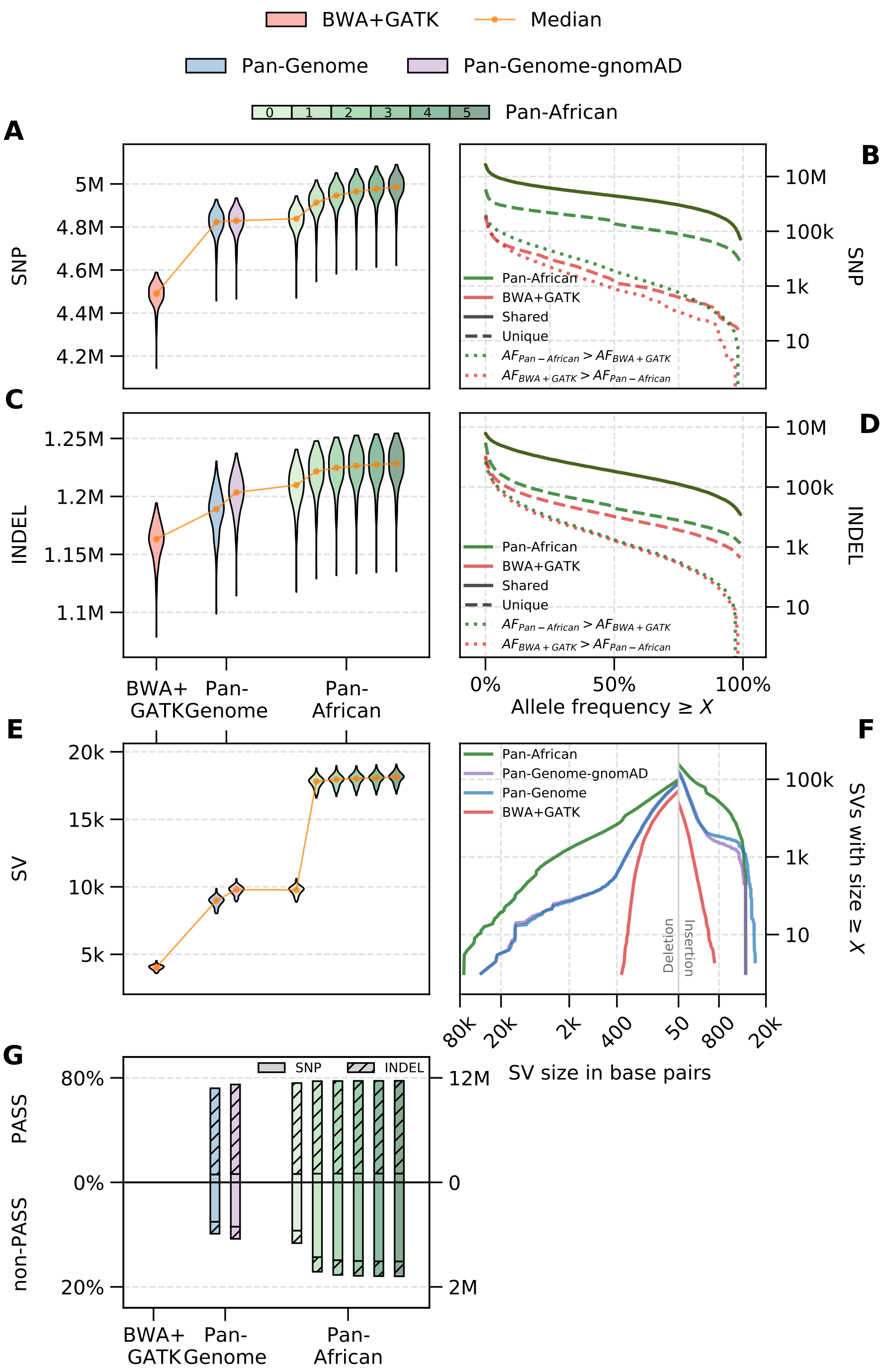

Supplementary Figure 9. Variant calling results for graphs including gnomAD Pan-Genome graph. Two-sided Wilcoxon tests between gnomAD Pan-Genome and other distributions are performed. In all cases except one (SV counts between gnomAD Pan-Genome and Pan-African-0) the difference is significant ($p < 10^{-24}$).

Supplementary Section 3 Alignment Statistics

Supplementary Section 3.1 Alignment QC

We determined the alignment quality of reads through commonly used metrics like the number of mapped reads, proper reads, mapping quality, error rate and coverage. We compared population-specific Pan-African graphs to Pan-Genome graph and linear reference based BWA+GATK workflow. Means of metrics over all benchmark results are presented in Supplementary

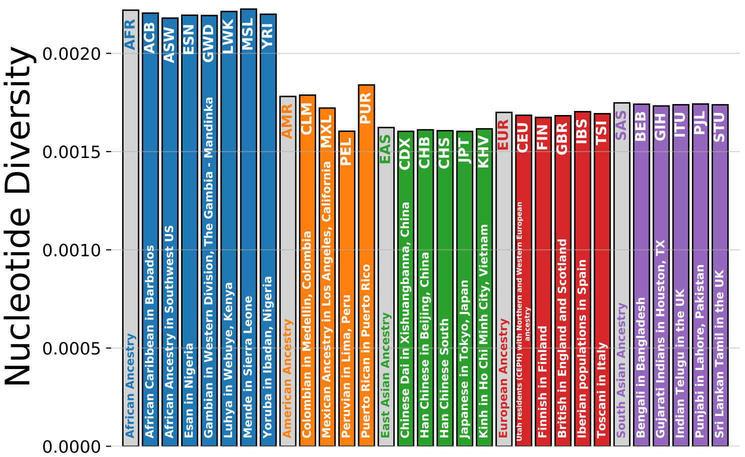

Supplementary Figure 10. Nucleotide diversity for all super-populations and populations in the 1000 Genomes dataset

Table 8. We observed that BWA reported more aligned reads, consistent with the more lenient approach. Graph workflows resulted with less improper reads and error rates. Decrease in improper reads is by nearly 5 million and error rate decreased by nearly half for the final population-specific graph. Also, we see that while BWA mapped on average 1.1 million more higher confidence reads (MAPQ$\geq$ 20), it includes 2.4 million more multi-mapped reads (MAPQ$=$ 0) and 5.5 million more low quality alignments (MAPQ$<$ 20). Additionally, graph generated directly from BWA+GATK variants instead of an iterative approach also shows a smaller range of error rate, improper reads and unmapped reads as well as less multi-maped and low quality reads and more high quality reads than linear BWA+GATK.

Population-specific graphs show improvment over Pan-Genome results. For instance, the number of mapped reads, high confidence reads and mean coverage increased in Pan-African iterations. Also, improper reads, low quality reads and error rate further decreased in Pan-African graph results. The number of multi-mapped reads initially increased in the first Pan-African graph but later graphs showed the decreasing trend.

Supplementary Section 3.2 Read Relocation Between Pipelines

Alignment results of the benchmark sample HG02470 are compared between BWA+GATK, Pan-Genome and Pan-African-5 workflows in order to get an insight on the quality of the reads that change locations. Reads that map to different locations are identified and analysed. Results are presented in Supplementary Table 9.

In the first table, reads that are aligned to the same chromosome with different positions are compared. Most notable difference is when the read alignment confidence changes. Pan-African-5 was able to identify more alignments with higher confidence (MAPQ $\geq$ 20) where BWA aligned with lower confidence (MAPQ $<$ 20). In the second table, similar comparison is performed but for read that map to different chromosomes or left unmapped. Pan-African-5 results in more unmapped reads compared to BWA. This is due to the lenient approach used by BWA compared to graph aligner for reporting read alignments by default. Another major difference is the number of low and high confidence reads. Graph aligner produces more high confidence reads compared to BWA and population-specific graphs further increases this compared to Pan-Genome.

Supplementary Section 4 Variant Statistics

Supplementary Section 4.1 Variant QC

Variants grouped by types are analyzed and shown in Supplementary Table 4. Graph pipelines called more variants compared to BWA+GATK. There is a 10.2% increase in average total variant counts for Pan-African-5 graph. Population-specific Pan-African graph resulted with additional 450,000 known SNPs and 6,041 known INDELs (with dbSNP v154) and additional 28,900 novel SNPs and 73,553 novel INDELs compared to BWA+GATK.

In order to understand the quality of these incoming variants, we analysed Het/Hom and Ti/Tv ratios which are well known to be population specific metrics for evolutionary reasons[10]. Het/Hom rate of known variants is around the expected 2.0 for all workflows. Novel SNP Het/Hom rates are higher for Pan-Genome graph than BWA+GATK but using Pan-African graphs lead to a small decrease. Still, there is an increase in novel INDEL Het/Hom rates using Pan-African graph. Ti/Tv rate appears to be very stable in the workflows, a small difference is observed in graph results, novel Ti/Tv rates are higher in graph results and

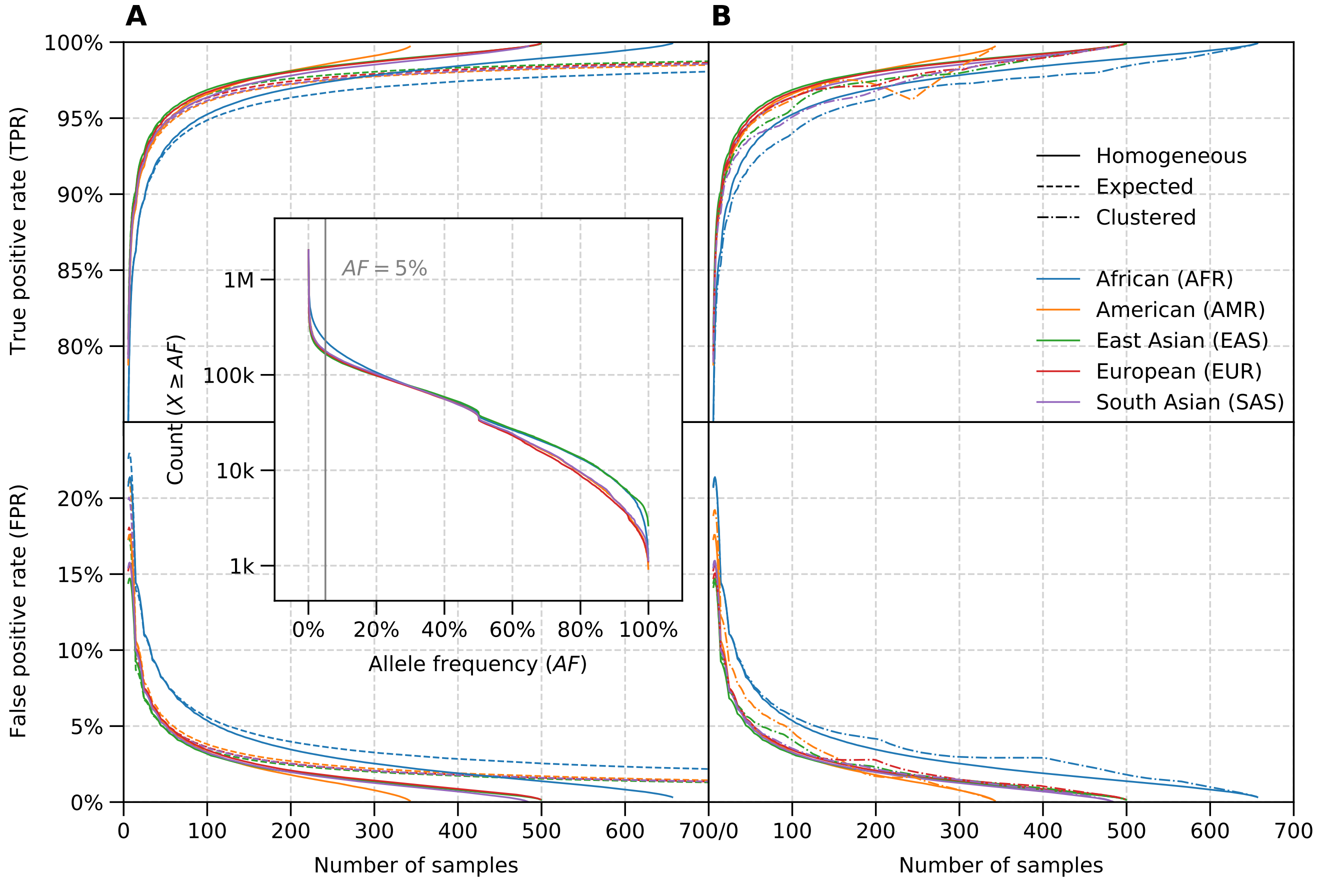

Supplementary Figure 11. True positive rate (TPR) and false positive rate (FPR) in population-specific graph references with respect to number of samples used for graph construction. TPR and FPR are calculated for each super-population shown in the legend. An allele frequency (AF) threshold of 5% is used, below which the variants are discarded. (a) Theoretical calculation (see Methods) and simulated results. (b) Simulated results with homogeneous and clustered sampling. Homogeneous sampling assumes samples are taken from populations uniformly, whereas clustered approach simulates the extreme case where samples are taken from each population until it is exhausted. Inset shows the AF distribution used for calculations, which is obtained empirically from the 1000 Genomes dataset for each major super-population.

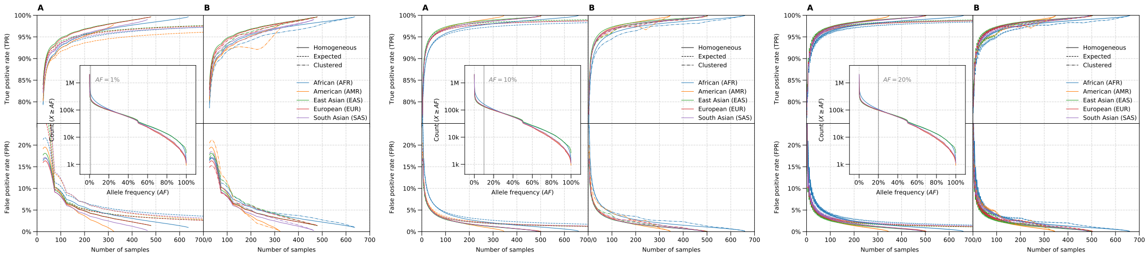

Supplementary Figure 12. TPR and FPR rates for different AF cutoffs (0.01, 0.1 and 0.2) for super-populations in 1000 Genomes data set

known Ti/Tv rate is slightly higher in BWA+GATK. Thus, both Het/Hom rate and Ti/Tv rate is quite similar in workflows indicating the quality of the variants is high. The non-iterative graph generated using BWA+GATK variants display a lower rate of Ti/Tv than linear BWA+GATK and higher rates of Het/Hom for all of the metrics separately.

Considering the total number of variants of samples from merged VCFs, Pan-African benchmarks, similar to per sample variants, include more SNPs and INDELs than BWA+GATK, Pan-African-5 resulted with 2.4 million more known and 606

thousand novel SNPs and 187 thousand more known and 1.9 million more novel INDELs than BWA+GATK. Singleton rates are approximately same, nearly 30% in Pan-African and 29.4% in BWA+GATK of known SNPs is singleton and 29% in Pan-African-5 and 23% in BWA+GATK of known INDELs is singleton. On the contrary, while the novel numbers are smaller than knowns, we observed that 67% in Pan-African-5 and 79.7% in BWA+GATK of novel SNPs is singleton. Novel INDEL singleton rates are smaller than SNPs, 27% in Pan-African-5 and 15.6% in BWA+GATK of novel INDELs is singleton. The graph generated using BWA+GATK graph includes more known but less novel SNPs and INDELs than Pan-African iteration graphs yet it includes more known but less novel singletons.

Based on the AF distributions, graph generated higher number of unique and common variants in higher frequencies. Furthermore, we noticed that, the number of known SNPs and INDELs decreases as the graph becomes more population specific comparing Pan-Genome to Pan-African graphs, and the number of novel SNPs and INDELs increases while the rate of singletons not changing too much. Ti/Tv rates for graph benchmarks are very generally similar but they are smaller than BWA+GATK Ti/Tv rates.

The number of total SNPs and INDELs for population-specific Pan-African graphs, Pan-Genome graph and BWA+GATK analysis are compared in difficult regions provided by Genome in a Bottle[11] and shown at Supplementary Table 13. The variants are mapped to the regions in two aspects: one as difficult regions vs the rest and second as low mapping and segmental duplications vs rest. We observed that in all regions, the graph pipeline is able to find more SNPs and more INDELs without any bias to any region. Both Pan-Genome and Pan-African graphs able to call more SNPs and INDELs than BWA+GATK in both difficult regions and low mapping and segmental duplication regions.

Original variant counts were shown without any filtration. In order to understand the effect of basic VCF filtering based on variant quality to the number of total variant calls with known vs novel distinctions, Het/Hom rates and Ti/Tv ratios, per sample VCF files were filtered with different QUAL (> 30, > 50 and > 70) values. Only the final graph (Pan-African-5) analyzed and compared to non-filtered metrics in this step. Details of this analysis can be found in Supplementary Table 12. We observed that the number of filtered variants is between 33,180 and 115,995 which is a small quantity of the total variant set (6,226,870). Yet, in filtered set of variants Het/Hom rate is decreased between 0.02 to 0.2 points and Ti/Tv rate is increased by 0.02 points. Furthermore, in merged set of variants the effect is sharper; we saw that the Ti/Tv ratio can be increased by 0.03 points for novel variants by filtering off only 298,706 variants.

Supplementary Section 4.2 Joint calling only regions

GATK team recommends joint analysis on a set of samples to perform variant discovery in their Best Practices workflow in order to empower population-wide information increasing sensitivity and accuracy over calling single samples. Since population-specific graphs found more frequent variants in the African super-population compared to GATK joint call results, we investigated whether graph can call the regions that are uniquely called by joint calling but not in single sample calls where they suggest population specific information is enriched.

We compared joint call results of the benchmark samples with GATK's single call results and counted those genotypes in two respects; homozygous (1/1 in joint call vs no call in single call) and heterozygous (0/1 in joint call vs no call in single call). Results are shown in Supplementary Table 6. We found that joint calling mostly rescues 0/1 genotypes (22,424,588 for 0/1 vs 1,713,068 for 1/1) and from these heterozygous genotypes, INDEL variant type is more than SNPs suggesting that single variant calling mostly loses heterozygous INDELs. Furthermore, most of these variants were tagged as PASS by VQSR. Conversely, most of the heterozygous SNPs rescued by joint calling (56,397) is tagged as non-PASS. Therefore, joint-calling is advantageous over single calling in heterozygous INDEL calling but not SNPs.

In our analysis, we hypothesized that population-specific graphs already include relevant variant information and could potentially identify these rescued sites. We found out that population-specific graphs can find most of the genotypes that were rescued by joint calling in samples. Population-specific graphs are able to find about 77% of 13,626,564 0/1 PASS genotypes and 86% of 1,461,641 1/1 PASS genotypes. In addition to that, we observed that most of the genotype missed using single call is INDEL type, and population-specific graphs able to identify 83% of 12,934,650 PASS INDELs while calling only 28% of 902,537 non-PASS INDELs. Out of 2,153,555 PASS SNPs, population-specific graphs successfully called 47% of them while calling only 17% of 8,146,914 non-PASS SNP regions. Finally, we confidently can say that population-specific graphs are more likely to identify joint-only regions that passed the VQSR filter. Pan-African graphs found nearly 78% of 15,088,205 PASS tagged regions while identifying only 18% of 9,049,451.

Finally, in order to asses the quality of these genotypes, we measured the genotype quality (GQ) and the likelihood difference between the two most likely genotypes (PL) in joint calling results. These two metrics are correlated with the variant caller's confidence on its genotype calls. Distributions of these confidence levels are shown in Figure 13 split by VQSR filter annotation PASS/non-PASS and type of variant (SNP/INDEL) (c.f. Figure 4g in the main text). For the PASS sites in SNPs, most of the higher confidence sites were genotyped in GRAF (green solid lines) while the missed sites (red solid lines) were mainly low confidence calls in joint calling. On the other hand, the sites that were filtered by VQSR (non-PASS, dashed lines)

are assigned lower confidence by joint calling, and GRAF is more stringent in genotyping variants at these loci. We observe similar trends for the INDELs.

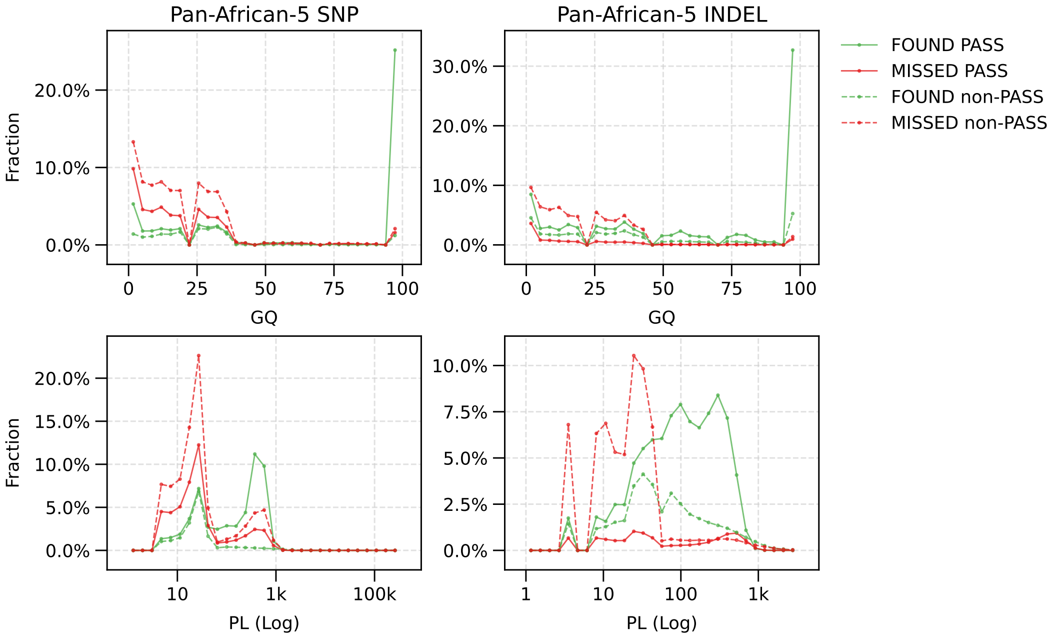

Supplementary Figure 13. Genotype quality (GQ) and likelihood difference between the two most likely genotypes (PL) distributions for sites that are identified by joint calling for SNP and INDELs. Results are stratified based on the PASS (solid line) or non-PASS (dashed line) as specified by VQSR and within that category whether (Found, green) or not (Missed, red) GRAF can genotype variants at those sites. Values are normalized according to the VQSR filter annotation for a variant type, such that sum of found (green line) and missed (red line) in each category is 100%.

Supplementary Section 4.3 Allele frequency distribution

We counted the number of SNPs and INDELs detected by GRAPH (with Pan-African-5) and the GATK and binned the counts by allele frequency (AF) in the bin size of 1% to investigate the sensitivity of the two pipelines. We counted the variant that are commonly and uniquely detected in both pipelines. We also divided commonly detected variants into to by the AF difference where in the first category the variants have higher AFs in GRAPH ($AF_{GRAF} > AF_{GATK}$) and in the second category the variants have higher AFs in GATK ($AF_{GATK} > AF_{GRAF}$). The cumulative distribution of these counts is given in Figure 14. The counts for this analysis are given in Supplementary Table 7.

Supplementary Section 4.4 Functional effect analysis

In order to evaluate the functional importance of the discovered variants, we used Ensembl Variant Effect Predictor[12] (VEP) GRCh38 release 101 on the unique GRAPH results with the Pan-African-5 graph reference and the unique GATK results that are generated by NYGC for the benchmark samples (N= 141). We used the `--flag_pick` option in VEP to select the annotation per transcript and used the picked annotation. We stratified the VEP results based on the IMPACT score defined by VEP (HIGH, MODERATE, LOW and MODIFIER), genomic region using the annotation definitions (exonic, intronic, intergenic) and allele frequency (AF) categories (Singleton where the variant is discovered in only single individual as heterozygous or homozygous, Rare where the variant is not a Singleton and has AF$<$ 5%, Common where the variant has AF$\geq$ 5%). The breakdown of the consequences of the VEP annotation is given in Supplementary Table 5.

Supplementary Section 5 Computational Efficiency Measurements

Execution details are summarized in the table 16. All executions are performed on a 72 core machine with 150 GB memory. Graph construction time shows the amount of time required to obtain the final graph used for sequencing sample data analysis. Graph and index loading time denotes the time it takes aligner to create graph along with search index from the input FASTA and VCF file. There is a slight correlation with the number of variants in the graph but total time is considerably fast (below 3 minutes). Graph and index memory usage is the aligner memory usage right after graph and index is constructed and before

alignment starts. It is consistently around 16GB with minor variations due to the variants included in the graph. Alignment time shows the mean duration for alignment of benchmark samples. Alignment time is affected by the number of variants as well as the composition of added variants. The jump at Pan-African 1 in alignment time mostly due to the addition of larger structural variants, specifically the insertions. Variant calling time follows the similar trend in alignment time, however the jump at Pan-African 1 is considerably smaller.

Supplementary Section 6 Bioinformatics pipelines and command lines

Supplementary Section 6.1 Seven Bridges Germline Pipeline

Input Files

- CRAM reference: GRCh38_full_analysis_set_plus_decoy_hla.fa
- CRAM reference index: GRCh38_full_analysis_set_plus_decoy_hla.fa.fai
- CRAM reference dictionary: GRCh38_full_analysis_set_plus_decoy_hla.dict
- Intervals: GRCh38.GRAF.Genome_Intervals.v1.bed
- Graph reference: GRCh38.GRAF.Reference.vcf.gz
- Graph reference index: GRCh38.GRAF.Reference.vcf.gz.tbi
- Linear reference: GRCh38.Linear.GRAF.Reference.fa
- Linear reference index: GRCh38.Linear.GRAF.Reference.fa.fai
- Input CRAM file: sample_name.cram
- Input CRAM file index: sample_name.cram.crai

Output Files

- Alignments: sample_name.bam
- Filtered variants: sample_name_filtered.vcf
- Variant stats: sample_name.stats
- Graph alignment summary: sample_name-summary.json
- Graph used edge data: sample_name.json.gz

Workflow Steps and Tools

- Read Processing: Samtools 1.9

```
samtools collate -u -O --threads 31 -n 64 --reference
  GRCh38_full_analysis_set_plus_decoy_hla.fa
  sample_name.cram |
  samtools view -F 2304 -o sample_name_collated.cram
  --reference GRCh38_full_analysis_set_plus_decoy_hla.fa
  --output-fmt CRAM --threads 31 -
```

- Alignment: Seven Bridges Graph Aligner 1.0rc3

```
aligner --cram_ref GRCh38_full_analysis_set_plus_decoy_hla.fa
  --vcf GRCh38.GRAF.Reference.vcf.gz
  --reference GRCh38.Linear.GRAF.Reference.fa
  --markdup --fmt bam
  -q sample_name_collated.cram
  --read_group_platform 'Illumina'
  --read_group_sample sample_name
  --read_group_library sample_name
```

```
--sort --tmp ./sort.chunk.XXXXXX
--sort_mem 30000 --hts_threads 40
--merge_threads 12 --keep
-o sample_name.bam &&
sambamba index -t 36 sample_name.bam
```

- Variant Calling: Seven Bridges Reassembly Variant Caller 1.0rc3

```
rasm -v sample_name.vcf -a GRCh38.GRAF.Genome_Intervals.v1.bed
  -f GRCh38.Linear.GRAF.Reference.fa -b sample_name.bam
  -g GRCh38.GRAF.Reference.vcf.gz -x all -s 10 -t 0
```

- Filtering: Bcftools 1.9

```
bcftools filter -o sample_name_filtered.vcf
  -e '(TYPE="snp" && (INFO/AD_Ratio[1] < 0.20 ||
  INFO/MBQ[1] < 15 || INFO/QD < 1 ||
  INFO/MQRankSum < -8 || INFO/FS > 50)) ||
  (TYPE="indel" && INFO/AD_Ratio[1] < 0.15)'
  -O v -s FP -m x sample_name.vcf
```

- Alignment and Variant stats: Graph Stats 1.0 & Bcftools 1.9

```
python graph_stats.py --bam sample_name.bam
  --vcf GRCh38.GRAF.Reference.vcf.gz --pretty

bash gzip_vcf.sh &&
  bcftools stats
  --fasta-ref GRCh38.Linear.GRAF.Reference.fa
  sample_name.vcf.gz > sample_name.stats;
  mkdir plots_dir &&
  plot-vcfstats sample_name.stats -p plots_dir/
```

Supplementary Section 6.2 Collect Benchmark Stats Pipeline

Input Files

- Annotation file: HG38.dbsnp154.vcf.gz
- Annotation file index: HG38.dbsnp154.vcf.gz.tbi
- Ploidy definition file:ploidy_hg38_vcffix.txt
- Reference sequence in FASTA format: GRCh38.Linear.GRAF.Reference.fa
- Reference sequence in FASTA format index: GRCh38.Linear.GRAF.Reference.fa.fai
- Samples list: sample_genders.txt
- Input variants file array: sample_name_1.vcf, sample_name_2.vcf ..

Output Files

- Filtered output VCF file: benchmark.vcf.gz
- Bcftools stat output file: benchmark.stats
- RTG vcfstats output file - known: benchmark.known.vcfstats
- RTG vcfstats output file - novel: benchmark.novel.vcfstats
- Counts: benchmark.frq.count
- Frequency: benchmark.frq
- HWE: benchmark.hwe
- Heterozygosity: benchmark.het

Workflow Steps and Tools

- Normalization: Bcftools 1.9

```
bgzip -c -f sample_name.vcf > sample_name.vcf.gz &&
  bcftools index -f -t sample_name.vcf.gz &&
  bcftools norm --threads 16
  --fasta-ref GRCh38.Linear.GRAF.Reference.fa
  --multiallelics -any --output sample_name.norm.vcf.gz
  --output-type z sample_name.vcf.gz
```

- Merging: Bcftools 1.9

```
bash gzip_vcf.sh &&
  bcftools merge  --output benchmark.merged.vcf.gz
  --threads 16 --missing-to-ref --merge none
  --output-type z
  [sample_name_1.norm.vcf.gz  sample_name_2.norm.vcf.gz ..]
```

- Annotations and Ploidy Fix: Bcftools 1.9 & VCFtools 0.1.14

```
bcftools index  -f -t benchmark.merged.vcf.gz &&
  bcftools annotate --output benchmark.merged.annotated.vcf
  --set-id . --remove "INFO" benchmark.merged.vcf.gz

outpbgzip -c -f benchmark.merged.annotated.vcf
  > benchmark.merged.annotated.vcf.gz &&
  bcftools index -f -t benchmark.merged.annotated.vcf.gz &&
  bcftools annotate --output benchmark.annotated.vcf
  --annotations HG38.dbsnp154.vcf.gz --columns ID
  --output-type v --remove "FILTER" --threads 16
  benchmark.merged.annotated.vcf.gz

cat benchmark.merged.annotated.vcf | vcf-fix-ploidy
  --fix-likelihoods --ploidy ploidy_hg38_vcffix.txt
  --samples sample_genders.txt > benchmark.annotated_fixed.vcf

bcftools +fill-tags -o benchmark.annotated_fixed.info.vcf
  -Ov benchmark.annotated_fixed.vcf
```

- Filtration: Bcftools 1.9

```
bgzip -c -f benchmark.annotated_fixed.info.vcf
  > benchmark.annotated_fixed.info.vcf.gz &&
  bcftools index -f -t benchmark.annotated_fixed.info.vcf.gz &&
  bcftools filter --output benchmark.vcf.gz --include 'AC>0'
  --output-type z --threads 16
  benchmark.annotated_fixed.info.vcf.gz
```

- Variant Statistics: Bcftools 1.9 & VCFtools 0.1.14 & RTG Tools 3.6.2

```
bash gzip_vcf.sh &&
  bcftools stats --split-by-ID --samples -
  --fasta-ref GRCh38.Linear.GRAF.Reference.fa
  benchmark.vcf.gz
  > benchmark.vcf.stats; mkdir plots_dir &&
  plot-vcfstats benchmark.stats -p plots_dir/
```

```
rtg vcfstats --novel benchmark.vcf.gz
  > benchmark.novel.vcfstats
rtg vcfstats --known benchmark.vcf.gz
  > benchmark.known.vcfstats

vcftools --counts2 --gzvcf benchmark.vcf.gz
  --out benchmark.frq.count
vcftools --het --gzvcf benchmark.vcf.gz
  --out benchmark.het
vcftools --hardy --gzvcf benchmark.vcf.gz
  --out benchmark.hwe
vcftools --freq2 --gzvcf benchmark.vcf.gz
  --out benchmark.frq
```

Supplementary Section 7 Supplementary tables

Required number of samples and corresponding false positive rates for target true positive rates and AF cut-offs.

		AFR		AMR		SAS		EUR		EAS	
AF Cut-off	TPR	Samples	FPR	Samples	FPR	Samples	FPR	Samples	FPR	Samples	FPR
0,01	%90	37	%31,18	37	%43,55	33	%41,05	32	%32,68	30	%33,27
0,01	%95	226	%6,30	375	%4,34	180	%7,56	174	%5,35	170	%5,21
0,01	%96	371	%4,67	674	%2,91	325	%4,82	315	%3,53	228	%5,01
0,01	%97	578	%4,02	1421	%1,66	576	%3,52	519	%2,89	470	%3,12
0,01	%98	1226	%2,78	3426	%0,73	1419	%2,09	937	%2,42	931	%2,37
0,01	%99	4074	%1,64	12184	%0,15	5317	%1,04	4270	%1,00	4065	%1,02
0,05	%90	25	%11,13	16	%11,87	15	%10,00	15	%9,65	15	%8,87
0,05	%95	96	%6,01	56	%5,49	47	%6,11	47	%6,17	46	%5,40
0,05	%96	156	%4,68	87	%4,52	77	%4,56	77	%4,62	67	%4,68
0,05	%97	314	%3,13	166	%3,07	156	%2,97	146	%3,10	117	%3,45
0,05	%98	684	%2,16	385	%1,95	365	%1,87	335	%1,96	304	%1,93
0,05	%99	2455	%1,21	1646	%0,92	1297	%1,03	1246	%1,03	1134	%1,00
0,1	%90	14	%16,56	9	%16,27	9	%15,13	9	%14,78	9	%13,89
0,1	%95	78	%5,39	34	%7,06	34	%6,55	39	%6,00	29	%6,84
0,1	%96	142	%3,74	68	%4,44	68	%4,12	68	%4,09	53	%4,51
0,1	%97	199	%3,46	137	%2,94	142	%2,71	142	%2,67	98	%3,32
0,1	%98	542	%1,94	268	%2,18	293	%2,00	273	%2,02	198	%2,34
0,1	%99	2032	%1,03	1019	%1,09	1203	%1,02	1172	%0,92	782	%1,13
0,2	%90	12	%15,36	12	%11,03	12	%11,24	12	%11,02	7	%13,86
0,2	%95	74	%4,99	49	%4,60	54	%4,68	49	%4,67	37	%5,30
0,2	%96	92	%4,66	79	%3,59	79	%3,89	74	%3,78	64	%3,70
0,2	%97	187	%3,16	112	%3,12	112	%3,43	107	%3,27	87	%3,31
0,2	%98	417	%2,05	277	%1,92	277	%2,17	274	%1,92	212	%2,07
0,2	%99	1722	%0,98	1329	%0,82	1174	%1,02	1094	%0,96	812	%1,05

TPR: True positive rate. Fraction of true variants in graph with respect to the ideal graph size
FPR: False positive rate. Fraction of false variants in graph with respect to the ideal graph size
Ideal graph: Graph constructed from all samples with respective AF cut-off.
Variants that are common between the graph constructed from subset of samples and ideal graph are considered true variants.
Extra variants in the subset graph that were not in ideal graph are considered false variants

Supplementary Table 1. Required number of samples and corresponding false positive rates for target true positive rates and AF cut-offs

Total graph edges and mean used edge counts per sample

	TOTAL EDGES						USED EDGES					
**Workflow**	SNP	MNP	INS	DEL	OTHER	TOTAL	SNP	MNP	INS	DEL	OTHER	TOTAL
**Non-iterative Pan-African**	9919205	10141	1609878	1702922	2720	13244866	4494069	6964	1337122	823103	1694	6667175
**Pan-Genome**	12500692	10158	818656	1131035	2895	14463436	4337206	7172	633737	391967	1920	5372002
**Pan-African-0**	9278644	10655	1404792	1492475	2957	12189523	4345684	7490	1184396	733881	1940	6273391
**Pan-African-1**	10583590	10144	1674791	1767556	2735	14038816	4862675	6935	1372664	845054	1642	7088971
**Pan-African-2**	11158974	10140	1763463	1843941	2728	14779246	5018916	6930	1433605	867760	1628	7328839
**Pan-African-3**	11522421	10142	1820040	1891888	2726	15247217	5108480	6925	1472150	881391	1623	7470570
**Pan-African-4**	11777384	10145	1860915	1926501	2728	15577673	5167509	6925	1499816	890976	1623	7566848
**Pan-African-5**	11967428	10141	1892349	1952936	2727	15825581	5209844	6918	1520818	898220	1619	7637419

OTHER type includes complex insertion and deletions like AT -> CGA or ATC -> G

Supplementary Table 2. Total graph edges and mean used edge counts per sample

Graph reference	Variant source	Number of variants in graph	Number of variants filtered out
**Pan-Genome**	GRCh38 alternative contigs	151.308	2.088
	1000 Genomes Project	13.386.980	34.820
	Simons Genome Diversity Project	10.444.099	25.704
	Indels discovered by Mills et al and 1000 Genomes consensus indels	1.411.165	2.249
	**Total**	**14.463.346**	**38.621**
**Pan-African-0**	GRCh38 alternative contigs	153.047	471
	Human Genome Structural Variation Consortium (HGSVC)	66.662	11.136
	Construction set 0 - Genome Aggragation Database v3 (gnomAD v3)	12.105.631	34
	**Total**	**12.248.698**	**11.629**
**Pan-African-1**	GRCh38 alternative contigs	150.890	2.506
	Human Genome Structural Variation Consortium (HGSVC)	64.599	13.199
	Construction set 0 - Genome Aggragation Database v3 (gnomAD v3)	12.072.468	33.197
	Construction set 1	12.847.183	58.918
	**Total**	**14.038.816**	**76.011**
**Pan-African-2**	GRCh38 alternative contigs	150.888	2.508
	Human Genome Structural Variation Consortium (HGSVC)	64.598	13.200
	Construction set 0 - Genome Aggragation Database v3 (gnomAD v3)	12.071.429	34.236
	Construction set 1	12.843.433	62.668
	Construction set 2	12.976.743	64.942
	**Total**	**14.779.246**	**89.251**
**Pan-African-3**	GRCh38 alternative contigs	150.886	2.510
	Human Genome Structural Variation Consortium (HGSVC)	64.504	13.294
	Construction set 0 - Genome Aggragation Database v3 (gnomAD v3)	12.070.734	34.931
	Construction set 1	12.841.845	64.256
	Construction set 2	12.974.399	67.286
	Construction set 3	13.041.292	68.605
	**Total**	**15.247.217**	**97.371**
**Pan-African-4**	GRCh38 alternative contigs	150.875	2.521
	Human Genome Structural Variation Consortium (HGSVC)	64.635	13.163
	Construction set 0 - Genome Aggragation Database v3 (gnomAD v3)	12.070.183	35.482
	Construction set 1	12.840.972	65.129
	Construction set 2	12.973.228	68.457
	Construction set 3	13.039.602	70.295
	Construction set 4	13.082.065	70.552
	**Total**	**15.577.673**	**102.463**
**Pan-African-5**	GRCh38 alternative contigs	150.863	2.533
	Human Genome Structural Variation Consortium (HGSVC)	64.836	12.962
	Construction set 0 - Genome Aggragation Database v3 (gnomAD v3)	12.069.944	35.721
	Construction set 1	12.840.510	65.591
	Construction set 2	12.972.566	69.119
	Construction set 3	13.038.695	71.202
	Construction set 4	13.080.679	71.938
	Construction set 5	13.080.199	71.517
	**Total**	**15.825.581**	**105.955**
**Non-iterative Pan-African**	GRCh38 alternative contigs	150.840	2.556
	Human Genome Structural Variation Consortium (HGSVC)	65.388	12.410
	Construction set 0 - Genome Aggragation Database v3 (gnomAD v3)	12.074.999	30.666
	NYGC BWA+GATK construction sets	12.112.609	29.389
	**Total**	**13.244.866**	**50.670**

Supplementary Table 3. Number of variants contributed by each source to the graphs

Mean variant statistics for 141 benchmark samples for each pipeline.																			
Pipeline	Type	SNP Het	SNP Hom	SNPs	Deletion Het	Deletion Hom	Deletions	Insertion Het	Insertion Hom	Insertions	INDEL Het	INDEL Hom	INDEL	Total Het	Total Hom	Total	Fraction	Transition	Transvertion
Linear (BWA+GATK)	Novel	19995	6898	27124	241045	91806	337820	273481	73353	352250	514525	165159	690070	534520	172056	717194	%12,69	14199	19823
	dbSNP154	2942802	1464282	4457198	199292	59456	261874	130651	79893	213455	329943	139348	475329	3272745	1603630	4932527	%87,31	3986647	1934833
Non-iterative Pan-African	Novel	14950	3888	19337	263434	89778	361399	305073	70586	383984	568507	160364	745382	583457	164252	764720	%12,69	9997	13228
	dbSNP154	3171017	1519098	4783471	195967	55178	256249	138995	79918	223919	334962	135096	480168	3505979	1654194	5263639	%87,31	4194789	2107780
Pan-Genome	Novel	17539	4227	22082	252195	92889	349780	294396	74134	373315	546590	167023	723095	564129	171251	745177	%12,39	11165	15145
	dbSNP154	3168997	1570235	4794623	195240	57496	255738	132401	82275	217539	327641	139771	473277	3496637	1710007	5267900	%87,61	4241269	2123589
Pan-African-0	Novel	17950	4293	22555	258593	92343	355693	307559	73505	386019	566151	165848	741712	584101	170141	764267	%12,63	11403	15445
	dbSNP154	3181898	1572344	4809788	197233	57407	257666	133642	82116	218628	330875	139523	476294	3512773	1711867	5286083	%87,37	4250710	2131422
Pan-African-1	Novel	24041	7492	32025	266433	93052	364420	314989	74135	394247	581423	167187	758666	605463	174679	790691	%12,87	17093	22423
	dbSNP154	3221579	1597601	4875807	198456	57744	259264	134702	82322	219937	333157	140066	479201	3554736	1737667	5355009	%87,13	4295023	2178386
Pan-African-2	Novel	29872	10245	40816	267270	93102	365332	316297	74123	395566	583567	167224	760898	613439	177470	801714	%12,97	22086	28975
	dbSNP154	3236481	1607782	4901330	198918	57922	259916	135081	82421	220426	333999	140343	480341	3570481	1748125	5381672	%87,03	4312391	2196721
Pan-African-3	Novel	34222	12360	47432	267736	93148	365860	317039	74147	396345	584775	167295	762205	618997	179654	809637	%13,05	25963	33828
	dbSNP154	3243838	1613076	4914208	199141	58005	260229	135202	82467	220599	334343	140472	480828	3578181	1753548	5395035	%86,95	4321435	2205848
Pan-African-4	Novel	37332	13990	52280	268048	93188	366219	317499	74151	396818	585547	167339	763037	622880	181329	815317	%13,11	28832	37438
	dbSNP154	3247629	1616215	4921262	199257	58066	260409	135317	82478	220728	334574	140544	481137	3582203	1756759	5402399	%86,89	4326480	2210997
Pan-African-5	Novel	39581	15390	56024	268274	93221	366486	317814	74146	397137	586088	167367	763623	625670	182757	819647	%13,16	30993	40421
	dbSNP154	3249998	1618341	4925853	199319	58088	260495	135429	82516	220875	334748	140604	481370	3584747	1758945	5407223	%86,84	4329872	2214323
INDEL= Insertions + Deletions																			
Novel set indicates the variants not found in dbSNP154 database																			

Supplementary Table 4. Mean variant statistics for 141 benchmark samples for each pipeline

Breakdown of the consequences identified for the variants that are unique to GRAPH (with Pan-African-5) and GATK using Ensembl Variant Effect Predictor GRCh38 release 101.								
Consequence	IMPACT	Region	Singleton (GRAPH)	Rare (GRAPH)	Common (GRAPH)	Singleton (GATK)	Rare (GATK)	Common (GATK)
frameshift_variant	HIGH	Exonic	688	139	51	368	281	188
start_lost	HIGH	Exonic	12	3	3	1	1	1
stop_gained	HIGH	Exonic	201	90	42	49	12	8
stop_lost	HIGH	Exonic	12	5	5	1	0	0
transcript_ablation	HIGH	Exonic	22	22	44	0	0	0
incomplete_terminal_codon_variant	LOW	Exonic	4	2	1	5	2	0
stop_retained_variant	LOW	Exonic	2	1	5	0	0	0
synonymous_variant	LOW	Exonic	2527	1695	1491	565	402	270
inframe_deletion	MODERATE	Exonic	490	226	58	147	124	30
inframe_insertion	MODERATE	Exonic	423	222	82	84	57	20
missense_variant	MODERATE	Exonic	4915	3123	2802	1128	841	474
protein_altering_variant	MODERATE	Exonic	27	11	2	12	6	1
3_prime_UTR_variant	MODIFIER	Exonic	15635	11778	4664	2767	3882	1313
5_prime_UTR_variant	MODIFIER	Exonic	2355	1435	644	433	505	132
coding_sequence_variant	MODIFIER	Exonic	9	3	2	4	1	0
non_coding_transcript_exon_variant	MODIFIER	Exonic	18782	15924	14893	2150	2561	1396
TFBS_ablation	MODERATE	Intergenic	458	389	206	100	102	17
TF_binding_site_variant	MODIFIER	Intergenic	8135	7407	5032	1170	1339	408
downstream_gene_variant	MODIFIER	Intergenic	174893	145172	80211	35961	44678	16167
intergenic_variant	MODIFIER	Intergenic	1132142	918265	633963	147982	184521	58128
regulatory_region_ablation	MODIFIER	Intergenic	18	52	75	1	1	1
regulatory_region_variant	MODIFIER	Intergenic	72536	58858	24242	16547	20690	6395
upstream_gene_variant	MODIFIER	Intergenic	187986	157551	88190	38699	49180	18326
splice_acceptor_variant	HIGH	Intronic	138	86	63	18	11	5
splice_donor_variant	HIGH	Intronic	159	132	95	21	20	12
splice_region_variant	LOW	Intronic	1310	982	730	159	171	133
intron_variant	MODIFIER	Intronic	1239286	998546	404136	255666	321315	92814

Supplementary Table 5. Breakdown of the consequences identified for the variants that are unique to GRAPH (with Pan-African-5) and GATK using Ensembl Variant Effect Predictor GRCh38 release 101

Total number of variants genotyped in each sample that were identified by joint calling only, stratified by genotype, variant type and VQSR filter.										
	The total number of genotypes per benchmarking sample									
	0/1				1/1				Total	
	SNP		INDEL		SNP		INDEL			
Workflow	PASS	non-PASS	PASS	non-PASS	PASS	non-PASS	PASS	non-PASS	PASS	non-PASS
**BWA+GATK (reference)**	2064394	7952015	11562170	846009	89161	194899	1372480	56528	15088205	9049451
**Non-iterative Pan-African**	978093	844563	9575378	192807	73828	40800	1187763	23427	11815062	1101597
**Pan-Genome**	847816	646195	8927780	182927	68069	37332	1054170	22247	10897835	888701
**Pan-African-0**	904393	794878	9389052	191492	70493	40895	1136685	23784	11500623	1051049
**Pan-African-1**	938493	1236070	9539089	222416	73178	59112	1170758	29204	11721518	1546802
**Pan-African-2**	942343	1286229	9551705	225061	73596	60961	1174806	29663	11742450	1601914
**Pan-African-3**	944602	1299512	9557449	226399	73799	61084	1176532	29715	11752382	1616710
**Pan-African-4**	945739	1304543	9561393	226707	73819	61254	1177890	29779	11758841	1622283
**Pan-African-5**	946671	1306862	9564290	227223	73943	61195	1178923	29828	11763827	1625108

Supplementary Table 6. Total number of variants genotyped in each sample that were indefitied by joint calling only

Comparison of BWA+GATK (GATK) to Pan-African 5 graph reference workflows in terms of Allele Frequencies in 0 to 100 bins for common or uqiue SNPs and INDELs.

AF bin (%)	Common SNP (GRAPH)	Common SNP (GATK)	Common INDEL (GRAPH)	Common INDEL (GATK)	Unique SNP (GRAPH)	Unique SNP (GATK)	Unique INDEL (GRAPH)	Unique INDEL (GATK)	AF_GRAPH>AF_GATK SNP	AF_GATK>AF_GRAPH SNP	AF_GRAPH>AF_GATK INDEL	AF_GATK>AF_GRAPH INDEL
0-1	10831396	10814211	1645096	1567625	1673009	202450	1907406	477921	167795	199580	362220	665389
1-2	3407642	3420885	830091	834648	435751	68103	467427	179641	61476	57926	128580	213236
2-3	1780239	1791212	479098	495497	185669	25104	179714	81910	31844	25465	57910	80533
3-4	1197545	1205749	338302	355355	104222	12798	96623	47295	20945	14619	34877	40740
4-5	896196	901979	265467	279273	72934	7994	62546	31533	15574	9850	23664	24467
5-6	709903	713662	219505	228735	56292	5501	44573	22431	12476	7188	17168	16116
6-7	409213	410862	131824	136849	32988	2732	24248	12129	7135	3724	9213	8792
7-8	513518	514496	169927	172706	41957	3254	28876	14107	8772	4278	10805	9209
8-9	440474	441010	149531	151537	37034	2568	22875	11025	7360	3522	8689	6817
9-10	384682	385514	131459	132810	33351	2221	18468	9277	6344	2808	6900	5464
10-11	340041	339946	118023	118612	29975	1776	15273	7590	5420	2098	5613	4380
11-12	299910	300206	105255	106297	27769	1616	12778	6437	4850	1913	4533	3630
12-13	277457	277153	97623	98439	24914	1523	11035	5655	4206	1598	3910	3071
13-14	167706	167224	59699	59937	15775	939	6507	3286	2675	935	2309	1843
14-15	235976	235290	82008	82786	22147	1248	8622	4499	3464	1157	2893	2262
15-16	217271	216418	75287	75908	20472	1076	7493	4043	3082	1127	2574	1983
16-17	199490	199015	69059	69543	19710	935	6601	3429	2803	843	2136	1698
17-18	184257	183792	63866	64066	18469	990	6012	3113	2548	848	1832	1537
18-19	174743	174051	60361	60939	17549	891	5379	2899	2297	742	1736	1411
19-20	109460	109010	37316	37677	11096	596	3270	1756	1413	435	971	774
20-21	153286	152829	51916	52692	15718	888	4591	2484	1932	579	1278	1093
21-22	143056	142802	48762	49111	15235	858	4210	2276	1766	556	1150	987
22-23	137056	136787	45104	45771	14324	1314	3773	2129	1597	543	1028	837
23-24	126549	126048	42032	42647	13923	634	3593	1909	1570	501	964	781
24-25	125385	124952	40556	40788	13817	703	3362	1795	1494	424	895	722
25-26	114928	114334	37231	37618	12819	682	2939	1624	1391	400	758	587
26-27	76771	76273	24435	24507	8523	692	1906	1051	758	256	467	392
27-28	108401	108074	34097	34321	12478	537	2747	1455	1177	344	611	502
28-29	103399	103021	31702	31951	11619	396	2426	1293	1135	310	572	512
29-30	95424	95038	30246	30141	11607	436	2398	1324	1088	295	523	459
30-31	96390	95211	29349	29679	11771	427	2238	1201	939	254	545	376
31-32	89927	89392	27134	27295	10368	403	2037	1105	890	212	451	352
32-33	86863	86681	26061	26146	10512	317	1923	1103	675	183	354	305
33-34	55636	55248	16796	16812	6803	279	1305	686	639	173	305	275
34-35	81044	80892	23799	23772	9624	340	1680	975	707	218	391	306
35-36	79112	78683	22449	22605	9539	303	1716	916	672	161	324	270
36-37	74836	74614	21645	21554	9125	513	1602	893	652	158	316	271
37-38	73771	73404	21004	21087	9418	354	1581	865	574	138	288	242
38-39	70118	69796	19736	19627	8895	355	1408	851	546	124	268	206
39-40	47085	46842	12903	13053	5851	205	959	538	345	76	174	152
40-41	65635	65321	17927	17921	8995	243	1291	718	530	114	248	191
41-42	64514	64139	17186	17319	8326	234	1242	623	525	101	204	192
42-43	63316	62998	16990	16927	8947	281	1198	611	476	89	202	163
43-44	62247	61985	16398	16325	9082	213	1246	602	473	105	193	186
44-45	61189	60884	15498	15561	9152	173	1221	607	426	85	174	156
45-46	57754	57364	14786	14691	9376	220	1203	540	402	93	179	173
46-47	39136	39008	9978	10097	6736	100	831	372	278	58	122	110
47-48	56811	56480	13976	13870	11281	176	1301	573	371	104	164	129
48-49	54341	54054	13350	13347	15320	185	1590	528	377	68	146	111
49-50	56011	54714	13607	13293	46380	349	3206	565	348	47	147	125
51-52	51906	51590	12561	12430	11433	99	1041	421	246	64	131	111
52-53	51888	51319	12207	12146	7791	76	796	448	229	45	120	112
53-54	33635	33381	7818	7731	4707	28	515	240	156	22	71	73
54-55	48208	47711	11179	11000	6078	47	707	387	184	42	84	101
55-56	48461	48073	10846	10805	5879	33	676	340	175	31	78	75
56-57	48640	48327	10688	10462	5607	29	592	349	197	22	81	67
57-58	47148	46745	10224	10138	5179	27	606	327	151	31	75	81
58-59	46544	46294	9889	9814	4598	34	553	282	157	26	78	85
59-60	44545	44248	9471	9397	4517	40	532	325	147	26	67	70
60-61	30751	30547	6281	6258	3045	46	352	191	62	13	45	37
61-62	44411	44065	9173	9047	4269	23	483	276	133	21	53	44
62-63	44528	43865	9009	8868	4361	30	515	301	132	83	61	62
63-64	42703	42457	8308	8200	4021	28	481	267	105	25	42	50
64-65	44063	43693	8273	8207	4064	39	446	248	100	23	51	44
65-66	42253	42000	8082	7942	3830	77	443	228	83	27	46	35
66-67	28094	27935	5272	5171	2663	47	292	171	76	12	37	37
67-68	42192	41935	7700	7693	3850	27	398	230	72	19	40	26
68-69	41401	41119	7307	7197	3907	42	374	245	81	23	41	40
69-70	40978	40760	7262	7176	3837	31	380	190	62	21	50	33
70-71	41179	40827	7132	7084	3742	36	372	249	67	41	34	30
71-72	40145	39867	6941	6848	3502	65	362	231	72	25	37	30
72-73	39703	39477	6672	6572	3364	30	353	181	64	14	40	34
73-74	27073	26996	4409	4419	2294	11	246	125	52	15	28	20
74-75	38295	38031	6309	6215	3489	40	315	186	62	12	24	32
75-76	38707	38526	6292	6232	3532	21	368	202	56	11	16	22
76-77	38174	38028	6045	5992	3261	23	303	159	57	5	31	35
77-78	38070	37805	6011	5917	3021	29	317	153	53	6	32	23
78-79	37382	37209	5988	5867	3432	13	309	187	40	10	28	24
79-80	37508	37298	5685	5613	3120	32	342	181	49	8	21	16
80-81	25412	25252	3802	3740	1939	8	203	104	23	3	14	12
81-82	37985	37789	5553	5502	3162	7	338	136	37	7	16	23
82-83	36643	36530	5346	5207	3172	14	321	175	42	6	18	11
83-84	36637	36407	5260	5203	3156	20	288	163	32	5	10	14
84-85	36655	36336	5153	5040	2838	19	262	164	21	2	18	14
85-86	36307	36106	5104	4972	2778	4	251	130	24	3	13	11
86-87	24860	24723	3400	3363	2053	20	185	112	19	1	6	13
87-88	36990	36843	4906	4816	2756	22	224	134	20	6	18	11
88-89	36015	35710	4839	4736	3026	7	260	121	22	2	13	10
89-90	36726	36501	4640	4608	2652	26	278	126	20	5	13	8
90-91	36120	36021	4384	4367	2730	17	254	156	17	5	9	8
91-92	35687	35628	4455	4362	2820	58	242	127	13	25	8	12
92-93	36500	36340	4312	4240	2905	12	248	113	9	1	8	6
93-94	25430	25390	3047	2986	1978	3	180	82	10	5	7	9
94-95	36667	36497	4191	4148	3431	4	245	108	16	3	10	5
95-96	37105	37050	4073	4048	3066	9	221	101	13	0	10	4
96-97	37884	37699	4028	3966	2760	8	251	109	6	0	3	4
97-98	39288	39242	4235	4249	3544	3	314	127	12	1	9	2
98-99	43891	43842	4544	4456	3903	7	333	142	11	1	5	7
99-100	39023	39054	3994	3991	3177	8	257	87	6	0	0	1
100	52570	51449	12468	12010	7765	19	1319	440	0	0	0	0

AF_GRAPH>AF_GATK: AF of GRAPH variant higher than GATK between commons

AF_GATK>AF_GRAPH: AF of GATK variant higher than GRAPH between commons

Supplementary Table 7. Comparison of BWA+GATK (GATK) to Pan-African 5 graph reference workflows in terms of Allele Frequencies in 0 to 100 bins for common or uqiue SNPs and INDELs

Mean alignment statistics for 141 benchmark samples for each workflow.

Workflow	Total Reads	Mapped Reads	Unmapped Reads	Mapped Bases -	Mapped Bases -	Proper Reads	Improper Reads	MAPQ=0	MAPQ<20	MAPQ>=20	Error Rate -	Error Rate -	Breadth Coverage	Mean Coverage
**BWA+GATK**	758773232	757672206	1101026	113600365312	-	738927751	18744455	36467090	47690168	709982038	0,00592	-	0,94136	34,26996
**Non-iterative Pan-African**	758773232	750507981	8265251	111793329498	111796468607	735819513	14688467	32859861	41490741	709017240	0,00627	0,00327	0,93991	34,00046
**Pan-Genome**	758773232	750140783	8632449	111812590808	111836979531	735531566	14609217	33473275	41948889	708191894	0,00502	0,00351	0,93979	33,99304
**Pan-African-0**	758773232	750326303	8446929	111868734834	111863053070	735126206	15200097	32991718	41726812	708599492	0,00552	0,00338	0,93978	34,02052
**Pan-African-1**	758773232	750679484	8093748	111863517308	111831736448	736473551	14205933	34414237	42201718	708477766	0,00675	0,00307	0,93924	34,12193
**Pan-African-2**	758773232	750785590	7987642	111881201340	111857262029	736707553	14078038	34154216	41952704	708832886	0,00699	0,00303	0,93893	34,16163
**Pan-African-3**	758773232	750809857	7963375	111871772617	111873851993	736845027	13964830	34102988	41907901	708901956	0,00706	0,00300	0,93874	34,18447
**Pan-African-4**	758773232	750821254	7951978	111852247382	111898073590	736914694	13906561	34030214	41860247	708961008	0,00711	0,00299	0,93862	34,19949
**Pan-African-5**	758773232	750830643	7942589	111870947148	111882464919	736959618	13871025	34069662	41915002	708915641	0,00715	0,00298	0,93853	34,21016

Notes:

MAPQ = Mapping Quality

Error Rate: Fraction of different bases against reference in all reads (mismatch/mapped bases)

Linear vs Graph reference

Supplementary Table 8. Mean alignment statistics for 141 benchmark samples for each workflow

Read relocations in between BWA and Pan-Genome, BWA and Pan-African 5 graph and Pan-Genome and Pan-African 5 graph BAM files within or between contigs for sample HG02470.

	Within Contigs								
		BAM1				BAM2			
**BAM1 vs BAM2**	**Total**	**MAPQ=0**	**MAPQ<20**	**BAM1 MAPQ=0 &**	**BAM1 MAPQ<20 &**	**MAPQ=0**	**MAPQ<20**	**BAM2 MAPQ=0 & BAM1**	**BAM2 MAPQ<20 & BAM1**
**BWA vs Pan-Genome**	36.296.024	18.162.741	2.362.023	871.640	586.731	18.155.558	1.938.040	698.159	329.046
**BWA vs Pan-African**	45.743.936	19.930.071	3.966.197	2.450.520	1.148.948	19.935.452	3.670.241	2.680.311	628.582
**Pan-Genome vs Pan-African**	30.592.522	14.807.288	3.496.077	2.199.645	485.995	15.194.652	3.537.697	2.306.062	808.562

	Between Contigs										
		BAM1					BAM2				
**BAM1 vs BAM2**	**Total**	**Unmapped**	**MAPQ=0**	**MAPQ<20**	**BAM1 MAPQ=0 &**	**BAM1 MAPQ<20 &**	**Unmapped**	**MAPQ=0**	**MAPQ<20**	**BAM2 MAPQ=0 & BAM1**	**BAM2 MAPQ<20 & BAM1**
**BWA vs Pan-Genome**	10.628.290	773	5.255.883	**1.968.009**	1.214.762	907.698	**6.882.798**	4.782.315	1.464.298	655.339	489.842
**BWA vs Pan-African**	13.615.284	230,53	6.258.467	**2.726.943**	2.126.631	1.413.298	**6.412.443**	5.606.908	1.773.352	1.326.339	608.440
**Pan-Genome vs Pan-African**	8.037.266	121.257	3.150.944	**1.029.761**	1.044.018	331.255	776.266	3.297.955	1.449.517	1.190.473	751.567

MAPQ: Mapping Quality

Supplementary Table 9. Read relocations in between BWA and Pan-Genome, BWA and Pan-African 5 graph and Pan-Genome and Pan-African 5 graph BAM files within or between contigs for sample HG02470

Mean variant ratios for 141 benchmark samples.

		Het/Hom					Ti/Tv
**Workflow**	**Type**	**SNP**	**Deletion**	**Insertion**	**INDEL**	**Total**	**SNP**
	**Novel**	2,90	2,63	3,73	3,12	3,10	0,72
	**dbSNP154**	2,01	3,56	1,64	2,37	2,04	2,06
**BWA+GATK**	**Total**	2,02	2,91	2,64	2,76	2,15	2,05
	**Novel**	3,87	2,93	4,32	3,54	3,55	0,76
	**dbSNP154**	2,09	3,55	1,74	2,48	2,12	1,99
**Non-iterative Pan-African**	**Total**	2,92	3,17	2,95	3,06	2,25	1,98
	**Novel**	4,16	2,72	3,98	3,28	3,30	0,74
	**dbSNP154**	2,02	3,40	1,61	2,35	2,05	2,00
**Pan-Genome**	**Total**	2,03	2,98	2,73	2,85	2,16	1,99
	**Novel**	4,19	2,80	4,20	3,42	3,44	0,74
	**dbSNP154**	2,03	3,44	1,63	2,37	2,05	1,99
**Pan-African-0**	**Total**	2,03	3,05	2,84	3,05	2,18	1,99
	**Novel**	3,21	2,87	4,25	3,48	3,47	0,76
	**dbSNP154**	2,02	3,44	1,64	2,38	2,05	1,97
**Pan-African-1**	**Total**	2,02	3,09	2,88	2,98	2,18	1,96
	**Novel**	3,21	2,87	4,25	3,48	3,46	0,76
	**dbSNP154**	2,02	3,44	1,64	2,38	2,04	1,96
**Pan-African-2**	**Total**	2,02	3,09	2,89	2,99	2,18	1,95
	**Novel**	2,77	2,88	4,28	3,50	3,45	0,77
	**dbSNP154**	2,01	3,44	1,64	2,38	2,04	1,96
**Pan-African-3**	**Total**	2,02	3,09	2,89	2,99	2,18	1,95
	**Novel**	2,77	2,88	4,28	3,50	3,45	0,77
	**dbSNP154**	2,01	3,44	1,64	2,38	2,04	1,96
**Pan-African-4**	**Total**	2,02	3,09	2,89	2,99	2,17	1,94
	**Novel**	2,57	2,88	4,29	3,51	3,43	0,77
	**dbSNP154**	2,01	3,43	1,64	2,38	2,04	1,96
**Pan-African-5**	**Total**	2,02	3,09	2,90	2,99	2,16	1,93

INDEL= Insertions + Deletions

Novel set identifed if not included in dbSNP154 database

Supplementary Table 10. Mean variant ratios for 141 benchmark samples

Variant counts in merged VCF files for 141 benchmark samples

	dbSNP-154			Novel		
	**SNPs**	**INDELs**	**Ti/Tv**	**SNPs**	**INDELs**	**Ti/Tv**
**BWA+GATK**	26982295	3378724	2,07	636318	3813959	0,92
**Non-iterative Pan-African**	29638398	3590558	1,99	1068397	5653107	0,86
**Pan-Genome**	29745187	3636523	1,98	1064531	5626471	0,85
**Pan-African-0**	29724853	3617093	1,98	1074415	5591375	0,85
**Pan-African-1**	29515163	3574290	1,99	1138379	5650360	0,85
**Pan-African-2**	29480823	3570150	1,99	1186150	5658201	0,84
**Pan-African-3**	29463118	3568471	2,00	1213112	5664061	0,84
**Pan-African-4**	29453613	3567445	2,00	1230420	5668781	0,84
**Pan-African-5**	29446788	3566478	2,00	1242915	5672168	0,84

Supplementary Table 11. Variant counts in merged VCF files for 141 benchmark samples

Per sample mean variant statistics for Pan-African-5 graph with different QUAL filtrations

Filter	Type	Deletion Het	Deletion Hom	Deletions	Insertion Het	Insertion Hom	Insertions	SNP Het	SNP Hom	SNPs	Total Het	Total Hom	Total	Transition	Transvertion
No Filter	Novel	268274	93221	366486	317814	74146	397137	39581	15390	56024	625670	182757	819647	30993	40421
	dbSNP154	199319	58088	260495	135429	82516	220875	3249998	1618341	4925853	3584747	1758945	5407223	4329872	2214323
QUAL>30	Novel	263974	93082	362001	312823	73993	391944	37110	14498	52628	613906	181573	806573	29251	37876
	dbSNP154	198393	58008	259480	134935	82416	220271	3235791	1614154	4907365	3569120	1754578	5387116	4318205	2203314
QUAL>50	Novel	257810	92860	355536	305490	73726	384259	35160	13558	49698	598460	180145	789493	27631	35625
	dbSNP154	197290	57905	258265	134443	82356	219716	3220847	1609228	4887357	3552580	1749489	5365338	4305120	2191464
QUAL>70	Novel	249924	92559	347237	295966	73377	374274	33457	12837	47243	579347	178773	768754	26320	33760
	dbSNP154	195983	57770	256807	133882	82272	219058	3204367	1604775	4866255	3534232	1744817	5342120	4291168	2179862

Per sample mean variant ratios for Pan-African-5 graph with different QUAL filtrations

		Het/Hom					Ti/Tv
Filter	Type	SNP	Deletion	Insertion	INDEL	Total	SNP
No Filter	Novel	2,5744	2,8801	4,2916	3,5052	3,4266	0,7665
	dbSNP154	2,0098	3,4347	1,6423	2,3825	2,0396	1,9554
	Total	2,0151	3,0929	2,8956	2,9925	2,1701	1,9341
QUAL>30	Novel	2,5626	2,8382	4,2329	3,4557	3,3842	0,7721
	dbSNP154	2,0062	3,4235	1,6384	2,3755	2,0358	1,9599
	Total	2,0112	3,0628	2,8651	2,9623	2,1622	1,9398
QUAL>50	Novel	2,5965	2,7785	4,1487	3,3847	3,3252	0,7753
	dbSNP154	2,0031	3,4105	1,6336	2,3669	2,0323	1,9645
	Total	2,0080	3,0212	2,8210	2,9193	2,1529	1,9455
QUAL>70	Novel	2,6091	2,7023	4,0384	3,2929	3,2436	0,7793
	dbSNP154	1,9984	3,3958	1,6285	2,3573	2,0272	1,9685
	Total	2,0032	2,9687	2,7640	2,8645	2,1402	1,9504

Number of variants and Ti/Tv rates in benchmark merged VCFs for different QUAL filters in Pan-African-5 graph

	dbSNP-154			Novel		
	SNPs	INDELs	Ti/Tv	SNPs	INDELs	Ti/Tv
No Filter	29446788	3566478	2,00	1242915	5672168	0,84
QUAL>30	29267020	3541739	2,01	1103008	5621107	0,86
QUAL>50	29115633	3518357	2,01	1010574	5568046	0,88
QUAL>70	28978856	3497433	2,02	944209	5513332	0,89

Supplementary Table 12. Per sample mean variant statistics for Pan-African-5 graph with different QUAL filtrations

Variant numbers for low mapping and segmental duplicational regions and difficult regions or the variants not in those regions.

	Not in lowmap and segdup regions								
	BWA+GATK	Non-iterative Pan-African	Pan-Genome	Pan-African-0	Pan-African-1	Pan-African-2	Pan-African-3	Pan-African-4	Pan-African-5
SNPs	25574552	26292759	26352645	26334279	26284785	26284066	26284807	26285218	26285049
INDELs	6686392	8466033	8467656	8419167	8453683	8455066	8456876	8459360	8461004
	Not in difficult regions								
	BWA+GATK	Non-iterative Pan-African	Pan-Genome	Pan-African-0	Pan-African-1	Pan-African-2	Pan-African-3	Pan-African-4	Pan-African-5
SNPs	21663455	21860534	21878622	21877023	21859671	21859729	21859994	21859948	21859850
INDELs	1211578	1476361	1445071	1441575	1475025	1474856	1474764	1474772	1475094
	Lowmap and segdup regions								
	BWA+GATK	Non-iterative Pan-African	Pan-Genome	Pan-African-0	Pan-African-1	Pan-African-2	Pan-African-3	Pan-African-4	Pan-African-5
SNPs	2044061	4413074	4456112	4464027	4367795	4381945	4390461	4397853	4403692
INDELs	550360	863925	863808	857978	856690	859298	861803	863014	863670
	Difficult regions								
	BWA+GATK	Non-iterative Pan-African	Pan-Genome	Pan-African-0	Pan-African-1	Pan-African-2	Pan-African-3	Pan-African-4	Pan-African-5
SNPs	5955158	8845299	8930135	8921283	8792909	8806282	8815274	8823123	8828891
INDELs	6007095	7840143	7852714	7799963	7820533	7824255	7828461	7832037	7834060
BED files used:									

https://ftp-trace.ncbi.nlm.nih.gov/ReferenceSamples/giab/release/genome-stratifications/v2.0/GRCh38/union/

Supplementary Table 13. Variant numbers for low mapping and segmental duplicational regions and difficult regions or the variants not in those regions

Case ID	Gender	Population Code	Population Name	Group
HG02470	male	ACB	African Caribbean	Benchmark
HG02484	male	ACB	African Caribbean	Benchmark
HG02496	male	ACB	African Caribbean	Benchmark
HG02439	male	ACB	African Caribbean	Benchmark
HG02455	male	ACB	African Caribbean	Benchmark
HG02477	female	ACB	African Caribbean	Benchmark
HG02481	male	ACB	African Caribbean	Benchmark
HG02501	male	ACB	African Caribbean	Benchmark
HG02502	female	ACB	African Caribbean	Benchmark
HG02505	female	ACB	African Caribbean	Benchmark
HG02536	male	ACB	African Caribbean	Benchmark
HG02541	male	ACB	African Caribbean	Benchmark
HG02545	male	ACB	African Caribbean	Benchmark
HG02546	female	ACB	African Caribbean	Benchmark
HG02549	female	ACB	African Caribbean	Benchmark
HG02554	male	ACB	African Caribbean	Benchmark
HG02555	female	ACB	African Caribbean	Benchmark
HG02557	male	ACB	African Caribbean	Benchmark
HG02558	female	ACB	African Caribbean	Benchmark
HG02577	female	ACB	African Caribbean	Benchmark
HG02580	female	ACB	African Caribbean	Benchmark
NA20357	female	ASW	African Ancestry SW	Benchmark
NA20359	female	ASW	African Ancestry SW	Benchmark
NA19922	male	ASW	African Ancestry SW	Benchmark
NA19923	female	ASW	African Ancestry SW	Benchmark
NA19984	male	ASW	African Ancestry SW	Benchmark
NA20298	male	ASW	African Ancestry SW	Benchmark
NA20339	female	ASW	African Ancestry SW	Benchmark
NA20351	male	ASW	African Ancestry SW	Benchmark
NA20412	female	ASW	African Ancestry SW	Benchmark
NA19913	female	ASW	African Ancestry SW	Benchmark
NA20274	female	ASW	African Ancestry SW	Benchmark
NA20318	male	ASW	African Ancestry SW	Benchmark
NA20320	female	ASW	African Ancestry SW	Benchmark
NA20321	female	ASW	African Ancestry SW	Benchmark
NA20355	female	ASW	African Ancestry SW	Benchmark
NA20362	male	ASW	African Ancestry SW	Benchmark
HG03166	male	ESN	Esan	Benchmark
HG03175	male	ESN	Esan	Benchmark
HG03189	female	ESN	Esan	Benchmark
HG03190	male	ESN	Esan	Benchmark
HG03193	male	ESN	Esan	Benchmark
HG03202	male	ESN	Esan	Benchmark
HG03265	male	ESN	Esan	Benchmark
HG03297	female	ESN	Esan	Benchmark
HG03298	male	ESN	Esan	Benchmark
HG03303	female	ESN	Esan	Benchmark
HG03304	male	ESN	Esan	Benchmark
HG03311	male	ESN	Esan	Benchmark
HG03313	male	ESN	Esan	Benchmark
HG03351	female	ESN	Esan	Benchmark
HG03352	male	ESN	Esan	Benchmark
HG03354	female	ESN	Esan	Benchmark
HG03363	female	ESN	Esan	Benchmark
HG03372	female	ESN	Esan	Benchmark
HG03499	female	ESN	Esan	Benchmark
HG03511	female	ESN	Esan	Benchmark
HG03517	female	ESN	Esan	Benchmark
HG03518	male	ESN	Esan	Benchmark
HG03520	female	ESN	Esan	Benchmark
HG03521	male	ESN	Esan	Benchmark
HG03024	male	GWD	Gambian Mandinka	Benchmark
HG03025	female	GWD	Gambian Mandinka	Benchmark
HG03240	male	GWD	Gambian Mandinka	Benchmark
HG03241	female	GWD	Gambian Mandinka	Benchmark
HG03246	male	GWD	Gambian Mandinka	Benchmark
HG03247	female	GWD	Gambian Mandinka	Benchmark
HG03538	male	GWD	Gambian Mandinka	Benchmark
HG03539	female	GWD	Gambian Mandinka	Benchmark
HG02819	male	GWD	Gambian Mandinka	Benchmark
HG02820	female	GWD	Gambian Mandinka	Benchmark
HG02854	male	GWD	Gambian Mandinka	Benchmark
HG02855	female	GWD	Gambian Mandinka	Benchmark
HG02568	female	GWD	Gambian Mandinka	Benchmark
HG02759	male	GWD	Gambian Mandinka	Benchmark
HG02760	female	GWD	Gambian Mandinka	Benchmark
HG02763	female	GWD	Gambian Mandinka	Benchmark
HG02870	female	GWD	Gambian Mandinka	Benchmark
HG02878	male	GWD	Gambian Mandinka	Benchmark
HG02879	female	GWD	Gambian Mandinka	Benchmark
HG02881	male	GWD	Gambian Mandinka	Benchmark
HG02882	female	GWD	Gambian Mandinka	Benchmark
HG02982	male	GWD	Gambian Mandinka	Benchmark
HG02983	female	GWD	Gambian Mandinka	Benchmark
NA19430	male	LWK	Luhya	Benchmark
NA19443	male	LWK	Luhya	Benchmark
NA19448	male	LWK	Luhya	Benchmark
NA19451	male	LWK	Luhya	Benchmark
NA19452	male	LWK	Luhya	Benchmark
NA19454	male	LWK	Luhya	Benchmark
NA19455	male	LWK	Luhya	Benchmark
NA19457	female	LWK	Luhya	Benchmark
NA19461	male	LWK	Luhya	Benchmark
NA19462	female	LWK	Luhya	Benchmark
NA19463	female	LWK	Luhya	Benchmark
NA19466	male	LWK	Luhya	Benchmark
NA19467	female	LWK	Luhya	Benchmark
NA19468	female	LWK	Luhya	Benchmark
NA19471	female	LWK	Luhya	Benchmark
NA19472	female	LWK	Luhya	Benchmark
NA19473	female	LWK	Luhya	Benchmark
NA19474	female	LWK	Luhya	Benchmark
NA19475	female	LWK	Luhya	Benchmark
HG03077	male	MSL	Mende	Benchmark
HG03079	female	MSL	Mende	Benchmark
HG03082	female	MSL	Mende	Benchmark
HG03086	female	MSL	Mende	Benchmark
HG03091	female	MSL	Mende	Benchmark
HG03095	female	MSL	Mende	Benchmark
HG03224	male	MSL	Mende	Benchmark
HG03225	male	MSL	Mende	Benchmark
HG03378	female	MSL	Mende	Benchmark
HG03382	male	MSL	Mende	Benchmark
HG03394	male	MSL	Mende	Benchmark
HG03432	male	MSL	Mende	Benchmark
HG03457	male	MSL	Mende	Benchmark
HG03558	female	MSL	Mende	Benchmark
HG03567	female	MSL	Mende	Benchmark
NA19238	female	YRI	Yoruba	Benchmark
NA19149	female	YRI	Yoruba	Benchmark
NA19184	male	YRI	Yoruba	Benchmark
NA19185	female	YRI	Yoruba	Benchmark
NA19189	male	YRI	Yoruba	Benchmark
NA19197	female	YRI	Yoruba	Benchmark
NA19214	female	YRI	Yoruba	Benchmark
NA19223	male	YRI	Yoruba	Benchmark
NA19235	female	YRI	Yoruba	Benchmark
NA19236	male	YRI	Yoruba	Benchmark
NA19247	female	YRI	Yoruba	Benchmark
NA19248	male	YRI	Yoruba	Benchmark
NA19256	male	YRI	Yoruba	Benchmark
NA18864	female	YRI	Yoruba	Benchmark
NA18865	male	YRI	Yoruba	Benchmark
NA18876	female	YRI	Yoruba	Benchmark
NA18877	male	YRI	Yoruba	Benchmark
NA18878	female	YRI	Yoruba	Benchmark
NA18879	male	YRI	Yoruba	Benchmark
NA18881	female	YRI	Yoruba	Benchmark
NA18915	male	YRI	Yoruba	Benchmark
NA19222	female	YRI	Yoruba	Benchmark
NA18874	male	YRI	Yoruba	Benchmark

Case ID	Gender	Population Code	Population Name	Group
HG01882	male	ACB	African Caribbean	Group 1
HG01883	female	ACB	African Caribbean	Group 1
HG01885	male	ACB	African Caribbean	Group 1
HG01956	female	ACB	African Caribbean	Group 1
HG02014	male	ACB	African Caribbean	Group 1
HG01886	female	ACB	African Caribbean	Group 1
HG01890	male	ACB	African Caribbean	Group 1
HG01889	female	ACB	African Caribbean	Group 1
HG02009	male	ACB	African Caribbean	Group 1
HG02010	female	ACB	African Caribbean	Group 1
HG01914	male	ACB	African Caribbean	Group 1
HG01915	female	ACB	African Caribbean	Group 1
HG01986	male	ACB	African Caribbean	Group 1
HG01958	female	ACB	African Caribbean	Group 1
HG02012	female	ACB	African Caribbean	Group 1
NA19625	female	ASW	African Ancestry SW	Group 1
NA19700	male	ASW	African Ancestry SW	Group 1
NA19701	female	ASW	African Ancestry SW	Group 1
NA19703	male	ASW	African Ancestry SW	Group 1
NA19704	female	ASW	African Ancestry SW	Group 1
NA19707	female	ASW	African Ancestry SW	Group 1
NA19711	male	ASW	African Ancestry SW	Group 1
NA19712	female	ASW	African Ancestry SW	Group 1
NA19818	male	ASW	African Ancestry SW	Group 1
HG02923	male	ESN	Esan	Group 1
HG02922	female	ESN	Esan	Group 1
HG02944	male	ESN	Esan	Group 1
HG02943	female	ESN	Esan	Group 1
HG02974	female	ESN	Esan	Group 1
HG02973	male	ESN	Esan	Group 1
HG03100	male	ESN	Esan	Group 1
HG03099	female	ESN	Esan	Group 1
HG03109	male	ESN	Esan	Group 1
HG03108	female	ESN	Esan	Group 1
HG03115	male	ESN	Esan	Group 1
HG03114	female	ESN	Esan	Group 1
HG03121	female	ESN	Esan	Group 1
HG03120	male	ESN	Esan	Group 1
HG03124	male	ESN	Esan	Group 1
HG02461	male	GWD	Gambian Mandinka	Group 1
HG02462	female	GWD	Gambian Mandinka	Group 1
HG02464	male	GWD	Gambian Mandinka	Group 1
HG02465	female	GWD	Gambian Mandinka	Group 1
HG02561	male	GWD	Gambian Mandinka	Group 1
HG02562	female	GWD	Gambian Mandinka	Group 1
HG02570	male	GWD	Gambian Mandinka	Group 1
HG02571	female	GWD	Gambian Mandinka	Group 1
HG02573	male	GWD	Gambian Mandinka	Group 1
HG02574	female	GWD	Gambian Mandinka	Group 1
HG02582	male	GWD	Gambian Mandinka	Group 1
HG02583	female	GWD	Gambian Mandinka	Group 1
HG02585	male	GWD	Gambian Mandinka	Group 1
HG02586	female	GWD	Gambian Mandinka	Group 1
HG02588	male	GWD	Gambian Mandinka	Group 1
HG02589	female	GWD	Gambian Mandinka	Group 1
HG02594	male	GWD	Gambian Mandinka	Group 1
HG02595	female	GWD	Gambian Mandinka	Group 1
NA19017	female	LWK	Luhya	Group 1
NA19019	female	LWK	Luhya	Group 1
NA19020	male	LWK	Luhya	Group 1
NA19023	female	LWK	Luhya	Group 1
NA19024	female	LWK	Luhya	Group 1
NA19025	male	LWK	Luhya	Group 1
NA19026	male	LWK	Luhya	Group 1
NA19027	male	LWK	Luhya	Group 1
NA19028	male	LWK	Luhya	Group 1
NA19030	female	LWK	Luhya	Group 1
NA19031	male	LWK	Luhya	Group 1
NA19035	male	LWK	Luhya	Group 1
NA19036	female	LWK	Luhya	Group 1
NA19037	female	LWK	Luhya	Group 1
NA19038	female	LWK	Luhya	Group 1
NA19042	female	LWK	Luhya	Group 1
HG03052	female	MSL	Mende	Group 1
HG03057	male	MSL	Mende	Group 1
HG03058	female	MSL	Mende	Group 1
HG03078	male	MSL	Mende	Group 1
HG03084	male	MSL	Mende	Group 1
HG03085	female	MSL	Mende	Group 1
HG03096	male	MSL	Mende	Group 1
HG03097	female	MSL	Mende	Group 1
HG03397	male	MSL	Mende	Group 1
HG03451	male	MSL	Mende	Group 1
HG03452	female	MSL	Mende	Group 1
HG03464	female	MSL	Mende	Group 1
HG03469	male	MSL	Mende	Group 1
HG03470	female	MSL	Mende	Group 1
NA18486	male	YRI	Yoruba	Group 1
NA18489	female	YRI	Yoruba	Group 1
NA18498	male	YRI	Yoruba	Group 1
NA18499	female	YRI	Yoruba	Group 1
NA18501	male	YRI	Yoruba	Group 1
NA18502	female	YRI	Yoruba	Group 1
NA18504	male	YRI	Yoruba	Group 1
NA18505	female	YRI	Yoruba	Group 1
NA18507	male	YRI	Yoruba	Group 1
NA18508	female	YRI	Yoruba	Group 1
NA18510	male	YRI	Yoruba	Group 1
NA18511	female	YRI	Yoruba	Group 1
NA18516	male	YRI	Yoruba	Group 1
NA18517	female	YRI	Yoruba	Group 1
NA18519	male	YRI	Yoruba	Group 1
NA18520	female	YRI	Yoruba	Group 1
NA18523	female	YRI	Yoruba	Group 1

Case ID	Gender	Population Code	Population Name	Group
HG01990	male	ACB	African Caribbean	Group 2
HG02053	male	ACB	African Caribbean	Group 2
HG02054	female	ACB	African Caribbean	Group 2
HG02255	male	ACB	African Caribbean	Group 2
HG02256	female	ACB	African Caribbean	Group 2
HG02314	male	ACB	African Caribbean	Group 2
HG02315	female	ACB	African Caribbean	Group 2
HG02281	male	ACB	African Caribbean	Group 2
HG02282	female	ACB	African Caribbean	Group 2
HG02317	male	ACB	African Caribbean	Group 2
HG02318	female	ACB	African Caribbean	Group 2
HG01912	male	ACB	African Caribbean	Group 2
HG01894	female	ACB	African Caribbean	Group 2
HG01880	female	ACB	African Caribbean	Group 2
HG01896	female	ACB	African Caribbean	Group 2
NA19713	female	ASW	African Ancestry SW	Group 2
NA19819	female	ASW	African Ancestry SW	Group 2
NA19834	male	ASW	African Ancestry SW	Group 2
NA19835	female	ASW	African Ancestry SW	Group 2
NA19900	male	ASW	African Ancestry SW	Group 2
NA19901	female	ASW	African Ancestry SW	Group 2
NA19904	male	ASW	African Ancestry SW	Group 2
NA19908	male	ASW	African Ancestry SW	Group 2
NA19909	female	ASW	African Ancestry SW	Group 2
HG03123	female	ESN	Esan	Group 2
HG03133	male	ESN	Esan	Group 2
HG03132	female	ESN	Esan	Group 2
HG03136	male	ESN	Esan	Group 2
HG03135	female	ESN	Esan	Group 2
HG03515	male	ESN	Esan	Group 2
HG03514	female	ESN	Esan	Group 2
HG03268	male	ESN	Esan	Group 2
HG03267	female	ESN	Esan	Group 2
HG03271	male	ESN	Esan	Group 2
HG03270	female	ESN	Esan	Group 2
HG03280	male	ESN	Esan	Group 2
HG03279	female	ESN	Esan	Group 2
HG03295	male	ESN	Esan	Group 2
HG03301	male	ESN	Esan	Group 2
HG02613	male	GWD	Gambian Mandinka	Group 2
HG02614	female	GWD	Gambian Mandinka	Group 2
HG02620	male	GWD	Gambian Mandinka	Group 2
HG02621	female	GWD	Gambian Mandinka	Group 2
HG02623	male	GWD	Gambian Mandinka	Group 2
HG02624	female	GWD	Gambian Mandinka	Group 2
HG02628	male	GWD	Gambian Mandinka	Group 2
HG02629	female	GWD	Gambian Mandinka	Group 2
HG02642	male	GWD	Gambian Mandinka	Group 2
HG02643	female	GWD	Gambian Mandinka	Group 2
HG02645	male	GWD	Gambian Mandinka	Group 2
HG02646	female	GWD	Gambian Mandinka	Group 2
HG02666	male	GWD	Gambian Mandinka	Group 2
HG02667	female	GWD	Gambian Mandinka	Group 2
HG02675	male	GWD	Gambian Mandinka	Group 2
HG02676	female	GWD	Gambian Mandinka	Group 2
HG02678	male	GWD	Gambian Mandinka	Group 2
HG02679	female	GWD	Gambian Mandinka	Group 2
NA19041	male	LWK	Luhya	Group 2
NA19043	male	LWK	Luhya	Group 2
NA19307	male	LWK	Luhya	Group 2
NA19308	male	LWK	Luhya	Group 2
NA19309	male	LWK	Luhya	Group 2
NA19310	female	LWK	Luhya	Group 2
NA19312	male	LWK	Luhya	Group 2
NA19314	female	LWK	Luhya	Group 2
NA19315	female	LWK	Luhya	Group 2
NA19316	female	LWK	Luhya	Group 2
NA19317	male	LWK	Luhya	Group 2
NA19320	female	LWK	Luhya	Group 2
NA19321	female	LWK	Luhya	Group 2
NA19323	female	LWK	Luhya	Group 2
NA19324	female	LWK	Luhya	Group 2
NA19327	female	LWK	Luhya	Group 2
HG03472	male	MSL	Mende	Group 2
HG03473	female	MSL	Mende	Group 2
HG03081	male	MSL	Mende	Group 2
HG03088	female	MSL	Mende	Group 2
HG03209	male	MSL	Mende	Group 2
HG03212	female	MSL	Mende	Group 2
HG03055	female	MSL	Mende	Group 2
HG03376	male	MSL	Mende	Group 2
HG03380	female	MSL	Mende	Group 2
HG03385	male	MSL	Mende	Group 2
HG03388	male	MSL	Mende	Group 2
HG03401	female	MSL	Mende	Group 2
HG03419	female	MSL	Mende	Group 2
HG03433	male	MSL	Mende	Group 2
NA18522	male	YRI	Yoruba	Group 2
NA18853	male	YRI	Yoruba	Group 2
NA18856	male	YRI	Yoruba	Group 2
NA18858	female	YRI	Yoruba	Group 2
NA18861	female	YRI	Yoruba	Group 2
NA18870	female	YRI	Yoruba	Group 2
NA18871	male	YRI	Yoruba	Group 2
NA18907	female	YRI	Yoruba	Group 2
NA18909	female	YRI	Yoruba	Group 2
NA18912	female	YRI	Yoruba	Group 2
NA18916	female	YRI	Yoruba	Group 2
NA19093	female	YRI	Yoruba	Group 2
NA19098	male	YRI	Yoruba	Group 2
NA19099	female	YRI	Yoruba	Group 2
NA19119	male	YRI	Yoruba	Group 2
NA19138	male	YRI	Yoruba	Group 2
NA19141	male	YRI	Yoruba	Group 2

Case ID	Gender	Population Code	Population Name	Group
HG01879	male	ACB	African Caribbean	Group 3
HG02013	male	ACB	African Caribbean	Group 3
HG01985	female	ACB	African Caribbean	Group 3
HG01988	male	ACB	African Caribbean	Group 3
HG01989	female	ACB	African Caribbean	Group 3
HG02051	male	ACB	African Caribbean	Group 3
HG02052	female	ACB	African Caribbean	Group 3
HG02095	female	ACB	African Caribbean	Group 3
HG02332	male	ACB	African Caribbean	Group 3
HG02107	male	ACB	African Caribbean	Group 3
HG02108	female	ACB	African Caribbean	Group 3
HG02111	female	ACB	African Caribbean	Group 3
HG02143	male	ACB	African Caribbean	Group 3
HG02144	female	ACB	African Caribbean	Group 3
HG02308	female	ACB	African Caribbean	Group 3
NA19914	female	ASW	African Ancestry SW	Group 3
NA19916	male	ASW	African Ancestry SW	Group 3
NA19917	female	ASW	African Ancestry SW	Group 3
NA19920	male	ASW	African Ancestry SW	Group 3
NA19921	female	ASW	African Ancestry SW	Group 3
NA19982	male	ASW	African Ancestry SW	Group 3
NA20126	male	ASW	African Ancestry SW	Group 3
NA20127	female	ASW	African Ancestry SW	Group 3
NA20276	female	ASW	African Ancestry SW	Group 3
HG03291	female	ESN	Esan	Group 3
HG03294	female	ESN	Esan	Group 3
HG03300	female	ESN	Esan	Group 3
HG03367	male	ESN	Esan	Group 3
HG03366	female	ESN	Esan	Group 3
HG02947	male	ESN	Esan	Group 3
HG02946	female	ESN	Esan	Group 3
HG02971	male	ESN	Esan	Group 3
HG02970	female	ESN	Esan	Group 3
HG02953	male	ESN	Esan	Group 3
HG02952	female	ESN	Esan	Group 3
HG02977	male	ESN	Esan	Group 3
HG03118	male	ESN	Esan	Group 3
HG03130	male	ESN	Esan	Group 3
HG03160	male	ESN	Esan	Group 3
HG02702	male	GWD	Gambian Mandinka	Group 3
HG02703	female	GWD	Gambian Mandinka	Group 3
HG02721	male	GWD	Gambian Mandinka	Group 3
HG02722	female	GWD	Gambian Mandinka	Group 3
HG02610	male	GWD	Gambian Mandinka	Group 3
HG02611	female	GWD	Gambian Mandinka	Group 3
HG02634	male	GWD	Gambian Mandinka	Group 3
HG02635	female	GWD	Gambian Mandinka	Group 3
HG02715	male	GWD	Gambian Mandinka	Group 3
HG02716	female	GWD	Gambian Mandinka	Group 3
HG02768	male	GWD	Gambian Mandinka	Group 3
HG02769	female	GWD	Gambian Mandinka	Group 3
HG02771	male	GWD	Gambian Mandinka	Group 3
HG02772	female	GWD	Gambian Mandinka	Group 3
HG02798	male	GWD	Gambian Mandinka	Group 3
HG02799	female	GWD	Gambian Mandinka	Group 3
HG02804	male	GWD	Gambian Mandinka	Group 3
HG02805	female	GWD	Gambian Mandinka	Group 3
NA19318	male	LWK	Luhya	Group 3
NA19319	male	LWK	Luhya	Group 3
NA19328	female	LWK	Luhya	Group 3
NA19331	male	LWK	Luhya	Group 3
NA19332	female	LWK	Luhya	Group 3
NA19334	male	LWK	Luhya	Group 3
NA19338	female	LWK	Luhya	Group 3
NA19346	male	LWK	Luhya	Group 3
NA19347	male	LWK	Luhya	Group 3
NA19350	male	LWK	Luhya	Group 3
NA19351	female	LWK	Luhya	Group 3
NA19355	female	LWK	Luhya	Group 3
NA19377	female	LWK	Luhya	Group 3
NA19378	female	LWK	Luhya	Group 3
NA19379	female	LWK	Luhya	Group 3
NA19390	female	LWK	Luhya	Group 3
HG03061	female	MSL	Mende	Group 3
HG03428	female	MSL	Mende	Group 3
HG03439	male	MSL	Mende	Group 3
HG03442	male	MSL	Mende	Group 3
HG03445	male	MSL	Mende	Group 3
HG03446	female	MSL	Mende	Group 3
HG03460	male	MSL	Mende	Group 3
HG03461	female	MSL	Mende	Group 3
HG03478	male	MSL	Mende	Group 3
HG03479	female	MSL	Mende	Group 3
HG03066	male	MSL	Mende	Group 3
HG03069	male	MSL	Mende	Group 3
HG03410	female	MSL	Mende	Group 3
HG03437	female	MSL	Mende	Group 3
NA19102	female	YRI	Yoruba	Group 3
NA19108	female	YRI	Yoruba	Group 3
NA19114	female	YRI	Yoruba	Group 3
NA19116	female	YRI	Yoruba	Group 3
NA19129	female	YRI	Yoruba	Group 3
NA19131	female	YRI	Yoruba	Group 3
NA19137	female	YRI	Yoruba	Group 3
NA19143	female	YRI	Yoruba	Group 3
NA19144	male	YRI	Yoruba	Group 3
NA19147	female	YRI	Yoruba	Group 3
NA19153	male	YRI	Yoruba	Group 3
NA19160	male	YRI	Yoruba	Group 3
NA19171	male	YRI	Yoruba	Group 3
NA19200	male	YRI	Yoruba	Group 3
NA19207	male	YRI	Yoruba	Group 3
NA19210	male	YRI	Yoruba	Group 3
NA19239	male	YRI	Yoruba	Group 3

Case ID	Gender	Population Code	Population Name	Group
HG02307	male	ACB	African Caribbean	Group 4
HG02283	male	ACB	African Caribbean	Group 4
HG02309	female	ACB	African Caribbean	Group 4
HG02284	male	ACB	African Caribbean	Group 4
HG02322	female	ACB	African Caribbean	Group 4
HG02323	male	ACB	African Caribbean	Group 4
HG02325	female	ACB	African Caribbean	Group 4
HG02337	female	ACB	African Caribbean	Group 4
HG02334	male	ACB	African Caribbean	Group 4
HG02330	male	ACB	African Caribbean	Group 4
HG02339	female	ACB	African Caribbean	Group 4
HG02343	male	ACB	African Caribbean	Group 4
HG02419	female	ACB	African Caribbean	Group 4
HG02427	female	ACB	African Caribbean	Group 4
HG02450	female	ACB	African Caribbean	Group 4
NA20278	male	ASW	African Ancestry SW	Group 4
NA20281	male	ASW	African Ancestry SW	Group 4
NA20282	female	ASW	African Ancestry SW	Group 4
NA20287	female	ASW	African Ancestry SW	Group 4
NA20289	female	ASW	African Ancestry SW	Group 4
NA20291	male	ASW	African Ancestry SW	Group 4
NA20294	female	ASW	African Ancestry SW	Group 4
NA20296	female	ASW	African Ancestry SW	Group 4
NA20340	male	ASW	African Ancestry SW	Group 4
HG02976	female	ESN	Esan	Group 4
HG03117	female	ESN	Esan	Group 4
HG03129	female	ESN	Esan	Group 4
HG03159	female	ESN	Esan	Group 4
HG03163	male	ESN	Esan	Group 4
HG03162	female	ESN	Esan	Group 4
HG03169	male	ESN	Esan	Group 4
HG03168	female	ESN	Esan	Group 4
HG03172	male	ESN	Esan	Group 4
HG03196	male	ESN	Esan	Group 4
HG03195	female	ESN	Esan	Group 4
HG03199	male	ESN	Esan	Group 4
HG03343	male	ESN	Esan	Group 4
HG03370	male	ESN	Esan	Group 4
HG02938	male	ESN	Esan	Group 4
HG02807	male	GWD	Gambian Mandinka	Group 4
HG02808	female	GWD	Gambian Mandinka	Group 4
HG02810	male	GWD	Gambian Mandinka	Group 4
HG02811	female	GWD	Gambian Mandinka	Group 4
HG02813	male	GWD	Gambian Mandinka	Group 4
HG02814	female	GWD	Gambian Mandinka	Group 4
HG02839	male	GWD	Gambian Mandinka	Group 4
HG02840	female	GWD	Gambian Mandinka	Group 4
HG02851	male	GWD	Gambian Mandinka	Group 4
HG02852	female	GWD	Gambian Mandinka	Group 4
HG02887	male	GWD	Gambian Mandinka	Group 4
HG02888	female	GWD	Gambian Mandinka	Group 4
HG02895	male	GWD	Gambian Mandinka	Group 4
HG02896	female	GWD	Gambian Mandinka	Group 4
HG03027	male	GWD	Gambian Mandinka	Group 4
HG03028	female	GWD	Gambian Mandinka	Group 4
HG03039	male	GWD	Gambian Mandinka	Group 4
HG03040	female	GWD	Gambian Mandinka	Group 4
NA19360	male	LWK	Luhya	Group 4
NA19372	male	LWK	Luhya	Group 4
NA19374	male	LWK	Luhya	Group 4
NA19375	male	LWK	Luhya	Group 4
NA19376	male	LWK	Luhya	Group 4
NA19380	male	LWK	Luhya	Group 4
NA19383	male	LWK	Luhya	Group 4
NA19391	female	LWK	Luhya	Group 4
NA19395	female	LWK	Luhya	Group 4
NA19399	female	LWK	Luhya	Group 4
NA19401	female	LWK	Luhya	Group 4
NA19403	female	LWK	Luhya	Group 4
NA19404	female	LWK	Luhya	Group 4
NA19431	female	LWK	Luhya	Group 4
NA19434	female	LWK	Luhya	Group 4
NA19435	female	LWK	Luhya	Group 4
HG03072	male	MSL	Mende	Group 4
HG03436	male	MSL	Mende	Group 4
HG03449	female	MSL	Mende	Group 4
HG03458	female	MSL	Mende	Group 4
HG03476	female	MSL	Mende	Group 4
HG03484	male	MSL	Mende	Group 4
HG03485	female	MSL	Mende	Group 4
HG03547	male	MSL	Mende	Group 4
HG03548	female	MSL	Mende	Group 4
HG03556	male	MSL	Mende	Group 4
HG03557	female	MSL	Mende	Group 4
HG03391	male	MSL	Mende	Group 4
HG03455	female	MSL	Mende	Group 4
HG03559	male	MSL	Mende	Group 4
NA19152	female	YRI	Yoruba	Group 4
NA19159	female	YRI	Yoruba	Group 4
NA19172	female	YRI	Yoruba	Group 4
NA19190	female	YRI	Yoruba	Group 4
NA19201	female	YRI	Yoruba	Group 4
NA19204	female	YRI	Yoruba	Group 4
NA19206	female	YRI	Yoruba	Group 4
NA19209	female	YRI	Yoruba	Group 4
NA19225	female	YRI	Yoruba	Group 4
NA18908	male	YRI	Yoruba	Group 4
NA18910	male	YRI	Yoruba	Group 4
NA18917	male	YRI	Yoruba	Group 4
NA18923	male	YRI	Yoruba	Group 4
NA18934	male	YRI	Yoruba	Group 4
NA19092	male	YRI	Yoruba	Group 4
NA19198	male	YRI	Yoruba	Group 4
NA19213	male	YRI	Yoruba	Group 4

Case ID	Gender	Population Code	Population Name	Group
HG02420	male	ACB	African Caribbean	Group 5
HG02429	male	ACB	African Caribbean	Group 5
HG02433	male	ACB	African Caribbean	Group 5
HG02442	male	ACB	African Caribbean	Group 5
HG02445	male	ACB	African Caribbean	Group 5
HG02489	male	ACB	African Caribbean	Group 5
HG02449	male	ACB	African Caribbean	Group 5
HG02471	female	ACB	African Caribbean	Group 5
HG02479	female	ACB	African Caribbean	Group 5
HG02485	female	ACB	African Caribbean	Group 5
HG02511	female	ACB	African Caribbean	Group 5
HG02497	female	ACB	African Caribbean	Group 5
HG02508	female	ACB	African Caribbean	Group 5
HG02537	female	ACB	African Caribbean	Group 5
HG02476	female	ACB	African Caribbean	Group 5
NA20299	female	ASW	African Ancestry SW	Group 5
NA20314	female	ASW	African Ancestry SW	Group 5
NA20317	female	ASW	African Ancestry SW	Group 5
NA20332	female	ASW	African Ancestry SW	Group 5
NA20334	female	ASW	African Ancestry SW	Group 5
NA20342	male	ASW	African Ancestry SW	Group 5
NA20346	male	ASW	African Ancestry SW	Group 5
NA20348	male	ASW	African Ancestry SW	Group 5
NA20356	male	ASW	African Ancestry SW	Group 5
HG03198	female	ESN	Esan	Group 5
HG03342	female	ESN	Esan	Group 5
HG03369	female	ESN	Esan	Group 5
HG02941	male	ESN	Esan	Group 5
HG02968	male	ESN	Esan	Group 5
HG02979	female	ESN	Esan	Group 5
HG02981	male	ESN	Esan	Group 5
HG03103	male	ESN	Esan	Group 5
HG03105	female	ESN	Esan	Group 5
HG03111	female	ESN	Esan	Group 5
HG03112	male	ESN	Esan	Group 5
HG03126	female	ESN	Esan	Group 5
HG03127	male	ESN	Esan	Group 5
HG03139	male	ESN	Esan	Group 5
HG03157	male	ESN	Esan	Group 5
HG03045	male	GWD	Gambian Mandinka	Group 5
HG03046	female	GWD	Gambian Mandinka	Group 5
HG03048	male	GWD	Gambian Mandinka	Group 5
HG03049	female	GWD	Gambian Mandinka	Group 5
HG03258	male	GWD	Gambian Mandinka	Group 5
HG03259	female	GWD	Gambian Mandinka	Group 5
HG02756	male	GWD	Gambian Mandinka	Group 5
HG02757	female	GWD	Gambian Mandinka	Group 5
HG02816	male	GWD	Gambian Mandinka	Group 5
HG02817	female	GWD	Gambian Mandinka	Group 5
HG02836	male	GWD	Gambian Mandinka	Group 5
HG02837	female	GWD	Gambian Mandinka	Group 5
HG02860	male	GWD	Gambian Mandinka	Group 5
HG02861	female	GWD	Gambian Mandinka	Group 5
HG02884	male	GWD	Gambian Mandinka	Group 5
HG02885	female	GWD	Gambian Mandinka	Group 5
HG02890	male	GWD	Gambian Mandinka	Group 5
HG02891	female	GWD	Gambian Mandinka	Group 5
NA19384	male	LWK	Luhya	Group 5
NA19385	male	LWK	Luhya	Group 5
NA19393	male	LWK	Luhya	Group 5
NA19394	male	LWK	Luhya	Group 5
NA19397	male	LWK	Luhya	Group 5
NA19428	male	LWK	Luhya	Group 5
NA19429	male	LWK	Luhya	Group 5
NA19436	female	LWK	Luhya	Group 5
NA19437	female	LWK	Luhya	Group 5
NA19438	female	LWK	Luhya	Group 5
NA19439	female	LWK	Luhya	Group 5
NA19440	female	LWK	Luhya	Group 5
NA19445	female	LWK	Luhya	Group 5
NA19446	female	LWK	Luhya	Group 5
NA19449	female	LWK	Luhya	Group 5
NA19456	female	LWK	Luhya	Group 5
HG03563	female	MSL	Mende	Group 5
HG03565	male	MSL	Mende	Group 5
HG03571	male	MSL	Mende	Group 5
HG03572	female	MSL	Mende	Group 5
HG03575	female	MSL	Mende	Group 5
HG03577	male	MSL	Mende	Group 5
HG03578	female	MSL	Mende	Group 5
HG03583	female	MSL	Mende	Group 5
HG03054	male	MSL	Mende	Group 5
HG03060	male	MSL	Mende	Group 5
HG03063	male	MSL	Mende	Group 5
HG03064	female	MSL	Mende	Group 5
HG03073	female	MSL	Mende	Group 5
HG03074	male	MSL	Mende	Group 5
NA19257	female	YRI	Yoruba	Group 5
NA18488	female	YRI	Yoruba	Group 5
NA18867	female	YRI	Yoruba	Group 5
NA18868	male	YRI	Yoruba	Group 5
NA18873	female	YRI	Yoruba	Group 5
NA18924	female	YRI	Yoruba	Group 5
NA18933	female	YRI	Yoruba	Group 5
NA19095	female	YRI	Yoruba	Group 5
NA19096	male	YRI	Yoruba	Group 5
NA19107	male	YRI	Yoruba	Group 5
NA19113	male	YRI	Yoruba	Group 5
NA19117	male	YRI	Yoruba	Group 5
NA19118	female	YRI	Yoruba	Group 5
NA19121	male	YRI	Yoruba	Group 5
NA19130	male	YRI	Yoruba	Group 5
NA19146	male	YRI	Yoruba	Group 5
NA19175	male	YRI	Yoruba	Group 5

Supplementary Table 14. List of samples that are used and their corresponding groups

Mean alignment statistics for 79 HGDP samples for each workflow.

Workflow	Total Reads	Mapped Reads	Unmapped	Mapped Bases -	Mapped Bases -	Proper Reads	Improper Reads	MAPQ=0	MAPQ<20	MAPQ>=20	Error Rate -	Error Rate -	Breadth	Mean Coverage
**BWA+GATK**	799013647	795314553	3699094	122121039959,379	122121039959,379	749481537	45833016	48873354	58267827	737046726	0,00838	0,00838	0,94311	36,2188
**Pan-Genome**	799013647	787215377	11798271	117273876435,151	117298664840,075	768714454	18500922	39841561	49541722	737673655	0,00730	0,00585	0,94126	35,8756
**Pan-African-5**	799013647	785906113	13107534	116910725053,974	117171496528,759	770574264	15331850	39274117	48037336	737868777	0,00936	0,00532	0,94003	36,1235

Notes:
MAPQ = Mapping Quality
Error Rate: Fraction of different bases against reference in all reads (mismatch/mapped bases)
Linear vs Graph reference

Mean variant statistics for 79 HGDP samples for each pipeline.

Pipeline	Type	SNP Het	SNP Hom	SNPs	Deletion Het	Deletion Hom	Deletions	Insertion Het	Insertion Hom	Insertions	INDEL Het	INDEL Hom	INDEL	Total Het	Total Hom	Total	Fraction	Transition	Transvertion
Linear (BWA+GATK)	Novel	29312	6029	36024	163641	45946	215399	186135	31134	222946	349776	77079	438345	379089	83109	474369	%8,82	19217	22837
	dbSNP154	2920273	1478119	4487406	175682	51090	231044	112471	70121	186448	288154	121212	417493	3208427	1599331	4904898	%91,18	4028408	1937117
Pan-Genome	Novel	47539	15335	64815	230745	88124	326288	272212	76053	356090	445241	153948	612569	473066	158675	645911	%12,39	36353	43796
	dbSNP154	3184779	1667260	4950413	188236	59810	253007	124677	83131	212489	305573	141819	456762	3419305	1760518	5284017	%87,61	4390712	2226960
Pan-African-5	Novel	27824	4728	33342	209461	85220	301380	235780	68728	311189	502957	164178	682378	550496	179513	747192	%12,12	17704	20365
	dbSNP154	3113732	1618699	4827255	183962	59040	247806	121611	82779	208956	312913	142941	465497	3497692	1810201	5415909	%87,88	4304715	2141239

Mean variant ratios for 79 HGDP samples.

		Het/Hom					Ti/Tv
Workflow	Type	SNP	Deletion	Insertion	INDEL	Total	SNP
BWA+GATK	Novel	4,82	3,65	6,33	4,70	4,71	0,83
	dbSNP154	1,98	3,45	1,61	2,38	2,01	2,08
	Total	1,99	3,53	2,97	3,24	2,14	2,07
Pan-Genome	Novel	5,97	2,46	3,43	2,89	2,98	0,86
	dbSNP154	1,93	3,12	1,47	2,16	1,95	2,01
	Total	1,94	2,73	2,36	2,54	2,03	2,00
Pan-African-5	Novel	3,11	2,62	3,59	3,07	3,07	0,83
	dbSNP154	1,91	3,15	1,50	2,19	1,94	1,97
	Total	1,92	2,84	2,50	2,66	2,04	1,95

Variant counts in merged VCF files for 79 HGDP samples

	dbSNP-154			Novel		
	SNPs	INDELs	Ti/Tv	SNPs	INDELs	Ti/Tv
**BWA+GATK**	25985799	2754075	2,05	1209113	3078038	1,14
**Pan-Genome**	27524467	3244960	2,01	1522744	5120888	1,04
**Pan-African-5**	27291094	3225360	2,01	1665507	5208641	1,01

79 HGDP African samples used in calculations

Case ID	Gender	Population Name
HGDP00450	male	Mbuti
HGDP00452	male	Biaka
HGDP00453	male	Biaka
HGDP00454	male	Biaka
HGDP00455	male	Biaka
HGDP00458	male	Biaka
HGDP00459	male	Biaka
HGDP00460	male	Biaka
HGDP00462	male	Mbuti
HGDP00463	male	Mbuti
HGDP00464	male	Biaka
HGDP00466	male	Biaka
HGDP00467	male	Mbuti
HGDP00469	male	Biaka
HGDP00470	male	Biaka
HGDP00471	female	Mbuti
HGDP00472	male	Biaka
HGDP00473	male	Biaka
HGDP00475	male	Biaka
HGDP00478	male	Mbuti
HGDP00479	male	Biaka
HGDP00904	male	Mandenka
HGDP00905	male	Mandenka
HGDP00906	male	Mandenka
HGDP00907	male	Mandenka
HGDP00908	male	Mandenka
HGDP00909	female	Mandenka
HGDP00910	female	Mandenka
HGDP00911	male	Mandenka
HGDP00912	male	Mandenka
HGDP00913	male	Mandenka
HGDP00914	female	Mandenka
HGDP00917	female	Mandenka
HGDP00918	female	Mandenka
HGDP00920	female	Yoruba
HGDP00924	female	Yoruba
HGDP00925	female	Yoruba
HGDP00926	female	Yoruba
HGDP00929	male	Yoruba
HGDP00930	male	Yoruba
HGDP00931	male	Yoruba
HGDP00933	female	Yoruba
HGDP00934	female	Yoruba
HGDP00935	female	Yoruba
HGDP00937	male	Yoruba
HGDP00938	female	Yoruba
HGDP00939	female	Yoruba
HGDP00940	male	Yoruba
HGDP00941	male	Yoruba
HGDP00942	male	Yoruba
HGDP00943	male	Yoruba
HGDP00944	male	Yoruba
HGDP00984	male	Mbuti
HGDP00985	male	Biaka
HGDP00986	male	Biaka
HGDP00992	male	San
HGDP00993	male	BantuSouthAfrica
HGDP00994	male	BantuSouthAfrica
HGDP01029	male	San
HGDP01031	male	BantuSouthAfrica
HGDP01033	male	BantuSouthAfrica
HGDP01081	male	Mbuti
HGDP01086	male	Biaka
HGDP01090	male	Biaka
HGDP01094	male	Biaka
HGDP01200	male	Mandenka
HGDP01201	female	Mandenka
HGDP01202	male	Mandenka
HGDP01283	male	Mandenka
HGDP01285	male	Mandenka
HGDP01405	male	BantuKenya
HGDP01406	male	BantuKenya
HGDP01408	male	BantuKenya
HGDP01411	male	BantuKenya
HGDP01412	male	BantuKenya
HGDP01415	male	BantuKenya
HGDP01416	male	BantuKenya
HGDP01418	male	BantuKenya
HGDP01419	male	BantuKenya

Supplementary Table 15. List of samples, alignment statistics and variant statistics for 79 HGDP samples for each workflow

Graph name	Graph construction	Graph and index loading time	Graph and index memory usage	Alignment time	Variant call time
PanGenome	1h 52m	164s	16.70 GB	2h 42m	41m
Pan-African 0	1h 34m	154s	16.01 GB	2h 54m	41m
Pan-African 1	2h 23m	164s	16.43 GB	4h 21m	46m
Pan-African 2	2h 41m	164s	16.71 GB	4h 31m	47m
Pan-African 3	2h 47m	173s	16.69 GB	4h 38m	47m
Pan-African 4	2h 54m	173s	16.73 GB	4h 41m	47m
Pan-African 5	3h 01m	174s	16.85 GB	4h 43m	47m

Supplementary Table 16. Execution details for graph, alignment and variant calling

References

1. Zook, J. M. *et al.* An open resource for accurately benchmarking small variant and reference calls. *Nature biotechnology* **37**, 561–566 (2019).
2. Karczewski, K. J. *et al.* The mutational constraint spectrum quantified from variation in 141,456 humans. *Nature* **581**, 434–443 (2020).
3. Rakocevic, G. *et al.* Fast and accurate genomic analyses using genome graphs. *Nature genetics* **51**, 354–362 (2019).
4. Consortium, . G. P. *et al.* A global reference for human genetic variation. *Nature* **526**, 68–74 (2015).
5. Mallick, S. *et al.* The simons genome diversity project: 300 genomes from 142 diverse populations. *Nature* **538**, 201–206 (2016).
6. Mills, R. E. *et al.* An initial map of insertion and deletion (indel) variation in the human genome. *Genome research* **16**, 1182–1190 (2006).
7. Ebert, P. *et al.* Haplotype-resolved diverse human genomes and integrated analysis of structural variation. *Science* (2021).
8. Byrska-Bishop, M. *et al.* High coverage whole genome sequencing of the expanded 1000 genomes project cohort including 602 trios. *bioRxiv* 2021.02.06.430068 (2021).
9. Bergström, A. *et al.* Insights into human genetic variation and population history from 929 diverse genomes. *Science* **367** (2020).
10. Wang, J., Raskin, L., Samuels, D. C., Shyr, Y. & Guo, Y. Genome measures used for quality control are dependent on gene function and ancestry. *Bioinformatics* **31**, 318–323 (2015).
11. Krusche, P. *et al.* Best practices for benchmarking germline small-variant calls in human genomes. *Nature biotechnology* **37**, 555–560 (2019).
12. McLaren, W. *et al.* The ensembl variant effect predictor. *Genome biology* **17**, 1–14 (2016).
