# Supplementary material for: Pan-African genome demonstrates how population-specific genome graphs improve high-throughput sequencing data analysis
Source: Nat Commun. 2022 Aug 4;13:4384. doi: 10.1038/s41467-022-31724-3 (PMC9352875; doi:10.1038/s41467-022-31724-3)
Supplement: Supplementary file 2 — Reporting Summary [file 41467_2022_31724_MOESM2_ESM.pdf]

nature portfolio

Corresponding author(s): Huseyin Serhat Tetikol

Last updated by author(s): Apr 29, 2022

Reporting Summary

Nature Portfolio wishes to improve the reproducibility of the work that we publish. This form provides structure for consistency and transparency in reporting. For further information on Nature Portfolio policies, see our Editorial Policies and the Editorial Policy Checklist.

Statistics

For all statistical analyses, confirm that the following items are present in the figure legend, table legend, main text, or Methods section.

n/a	Confirmed	
☐	☒	The exact sample size (n) for each experimental group/condition, given as a discrete number and unit of measurement
☐	☒	A statement on whether measurements were taken from distinct samples or whether the same sample was measured repeatedly
☐	☒	The statistical test(s) used AND whether they are one- or two-sided<br>*Only common tests should be described solely by name; describe more complex techniques in the Methods section.*
☒	☐	A description of all covariates tested
☒	☐	A description of any assumptions or corrections, such as tests of normality and adjustment for multiple comparisons
☒	☐	A full description of the statistical parameters including central tendency (e.g. means) or other basic estimates (e.g. regression coefficient) AND variation (e.g. standard deviation) or associated estimates of uncertainty (e.g. confidence intervals)
☐	☒	For null hypothesis testing, the test statistic (e.g. F, t, r) with confidence intervals, effect sizes, degrees of freedom and P value noted<br>*Give P values as exact values whenever suitable.*
☒	☐	For Bayesian analysis, information on the choice of priors and Markov chain Monte Carlo settings
☒	☐	For hierarchical and complex designs, identification of the appropriate level for tests and full reporting of outcomes
☒	☐	Estimates of effect sizes (e.g. Cohen's d, Pearson's r), indicating how they were calculated

Our web collection on statistics for biologists contains articles on many of the points above.

Software and code

Policy information about availability of computer code

Data collection	No software was used to collect data in this study. The data used in this study was collected and made available to public by other researchers, as referenced in the Data Availability section.
Data analysis	All software tools used in this study are listed with versions in the Supplementary Material along with the exact command lines used. A list of tools used can also be found below:<br>Seven Bridges Graph Aligner 1.0rc3<br>Seven Bridges Reassembly Variant Caller 1.0rc3<br>Samtools 1.9<br>Bcftools 1.9<br>VCFtools 0.1.14<br>RTG Tools 3.6.2

For manuscripts utilizing custom algorithms or software that are central to the research but not yet described in published literature, software must be made available to editors and reviewers. We strongly encourage code deposition in a community repository (e.g. GitHub). See the Nature Portfolio guidelines for submitting code & software for further information.

Data

Policy information about availability of data

All manuscripts must include a data availability statement. This statement should provide the following information, where applicable:

- Accession codes, unique identifiers, or web links for publicly available datasets
- A description of any restrictions on data availability
- For clinical datasets or third party data, please ensure that the statement adheres to our policy

The public high coverage dataset from 1000 Genomes project is available at https://www.internationalgenome.org/data-portal/data-collection/30x-grch38 . The public structural variation dataset from Human Genome Structural Variation Consortium is available at https://www.internationalgenome.org/data-portal/data-collection/hgsvc2 . The public gnomAD v3 dataset is available at https://gnomad.broadinstitute.org/downloads . The public sequencing sample dataset from the Human Genome Diversity Project is available at https://www.internationalgenome.org/data-portal/data-collection/hgdp . The Genome in a Bottle benchmarking samples are available at https://www.nist.gov/programs-projects/genome-bottle . The linear reference is based on GRCh38 patch 13 (available at https://www.ncbi.nlm.nih.gov/assembly/GCF_000001405.39) with decoy sequences hs38d1 (available at https://www.ncbi.nlm.nih.gov/assembly/GCA_000786075.2) and Epstein-Barr virus (available at https://www.ncbi.nlm.nih.gov/assembly/GCF_002402265.1). The Pan-Genome and Pan-African graph references are immediately available to all academic researchers through the public files repository of the NHLBI BioData Catalyst and can be made available on request for academic researchers on all other Seven Bridges cloud platforms (restrictions apply for commercial use, please contact Seven Bridges for terms and other details). To access the Pan-Genome and Pan-African graphs on the NHLBI BioData Catalyst, researchers must make a free account using their eRA Commons credentials (available through the US National Institute of Health) and then navigate to the public file repository at https://platform.sb.biodatacatalyst.nhlbi.nih.gov/resources/public-gallery/files . Any academic researchers who do not wish to access the files through the NHLBI BioData Catalyst may email support@sevenbridges.com and our 24/7 helpdesk team will respond with a target response time under 24h with an alternative access approach tailored to the user's request. Requestors will be asked to verify that their use is for academic purposes only. Source Data are provided with this paper.

Field-specific reporting

Please select the one below that is the best fit for your research. If you are not sure, read the appropriate sections before making your selection.

- [x] Life sciences
- [ ] Behavioural & social sciences
- [ ] Ecological, evolutionary & environmental sciences

For a reference copy of the document with all sections, see nature.com/documents/nr-reporting-summary-flat.pdf

Life sciences study design

All studies must disclose on these points even when the disclosure is negative.

Sample size	The sample size was not determined for this study. All relevant data in the referenced public datasets were used.
Data exclusions	No data was excluded from the analyses.
Replication	All findings can be reproduced using the same software tools and computer code used in this study.
Randomization	The samples were split into experimental groups such that each group encompassed all African subpopulations and contained the same ratio from of each subpopulation and sex (male/female). Aside from these restrictions, the samples were assigned pseudo-randomly to each experimental group. The exact sample list of each group is provided in the Supplementary Material.
Blinding	N/A. Benchmarking of different analysis approaches (standard BWA-GATK, Pan-Genome graph and Pan-African graph) was conducted on exactly the same sample set, providing consistent measurements of performance and eliminating any undesired bias in sample selection that would occur if benchmarking sets for each approach was different.

Reporting for specific materials, systems and methods

We require information from authors about some types of materials, experimental systems and methods used in many studies. Here, indicate whether each material, system or method listed is relevant to your study. If you are not sure if a list item applies to your research, read the appropriate section before selecting a response.

Materials & experimental systems

n/a	Involved in the study	
x		Antibodies
x		Eukaryotic cell lines
x		Palaeontology and archaeology
x		Animals and other organisms
x		Human research participants
x		Clinical data
x		Dual use research of concern

Methods

n/a	Involved in the study	
x		ChIP-seq
x		Flow cytometry
x		MRI-based neuroimaging
